# Correction: SYBR Gold dye enables preferential labelling of mitochondrial nucleoids and their time-lapse imaging by structured illumination microscopy

**DOI:** 10.1371/journal.pone.0321610

**Published:** 2025-04-01

**Authors:** The PLOS One Staff

## Notice of Republication

This article was republished on March 10, 2025, to correct errors in the figures and captions. that were introduced during the typesetting process. The publisher apologizes for the errors. Please download this article again to view the correct version. The originally published, uncorrected article and the republished, corrected articles are provided here for reference.

## Supporting Information

S1 File Originally published, uncorrected article. (PDF)

S2 File Republished, corrected article. (PDF)
