# Supplementary figures and images for: SYBR Gold dye enables preferential labelling of mitochondrial nucleoids and their time-lapse imaging by structured illumination microscopy
Source: PLoS One. 2018 Sep 18;13(9):e0203956. doi: 10.1371/journal.pone.0203956 (PMC6143240; doi:10.1371/journal.pone.0203956)

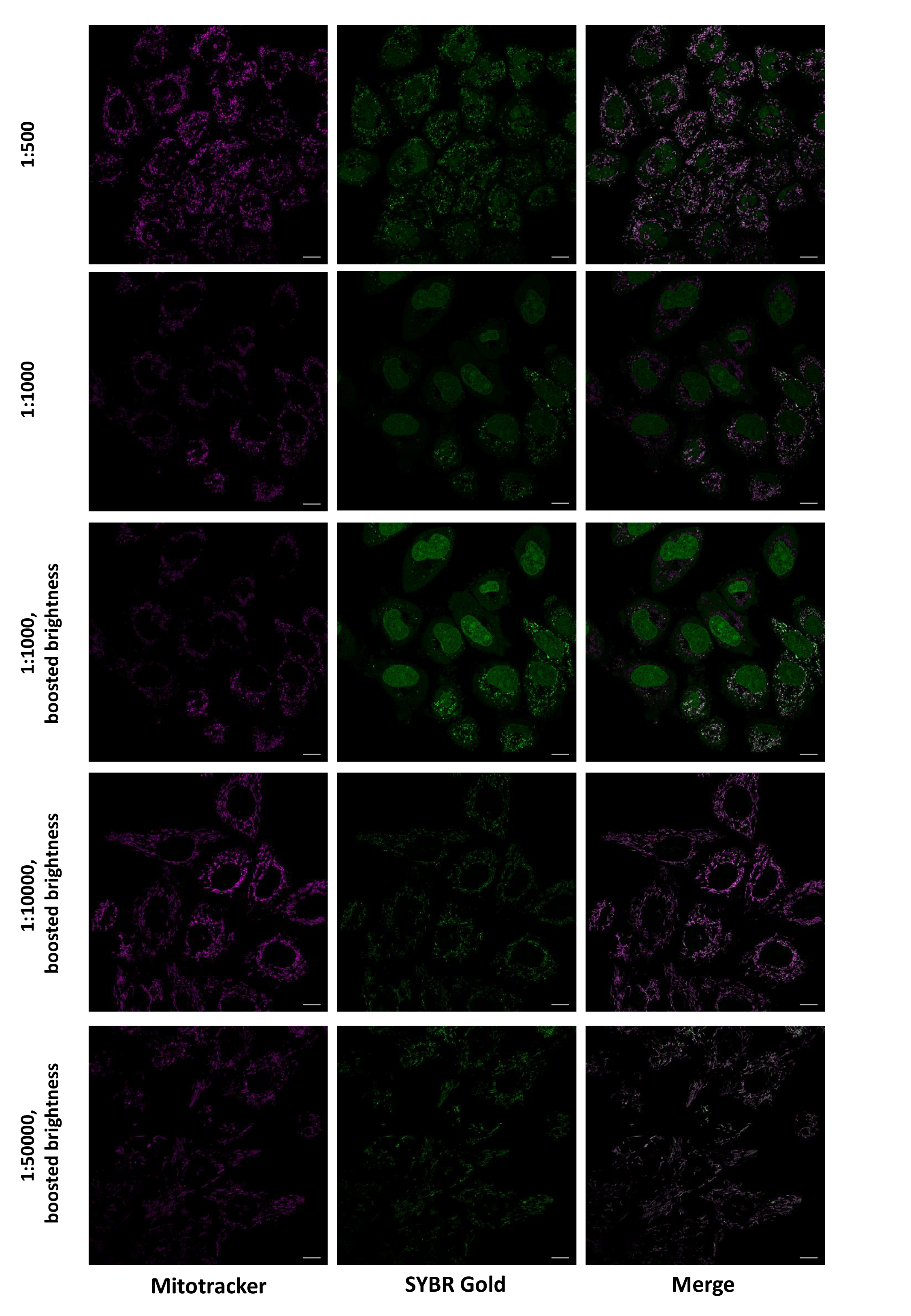

Supplement: S1 Fig — HeLa cells were incubated for 30 min with mixture of 0.25 μM Mitotracker CMXRos Red and indicated SYBR Gold dilution; the solution was replaced with DMEM and images were acquired on LSM880 Airyscan microscope, 63x 1.4 oil objective, sequential acquisition of color channels; Single optical slices are shown; scale bar 10 μm. (TIF) [file pone.0203956.s003.tif]

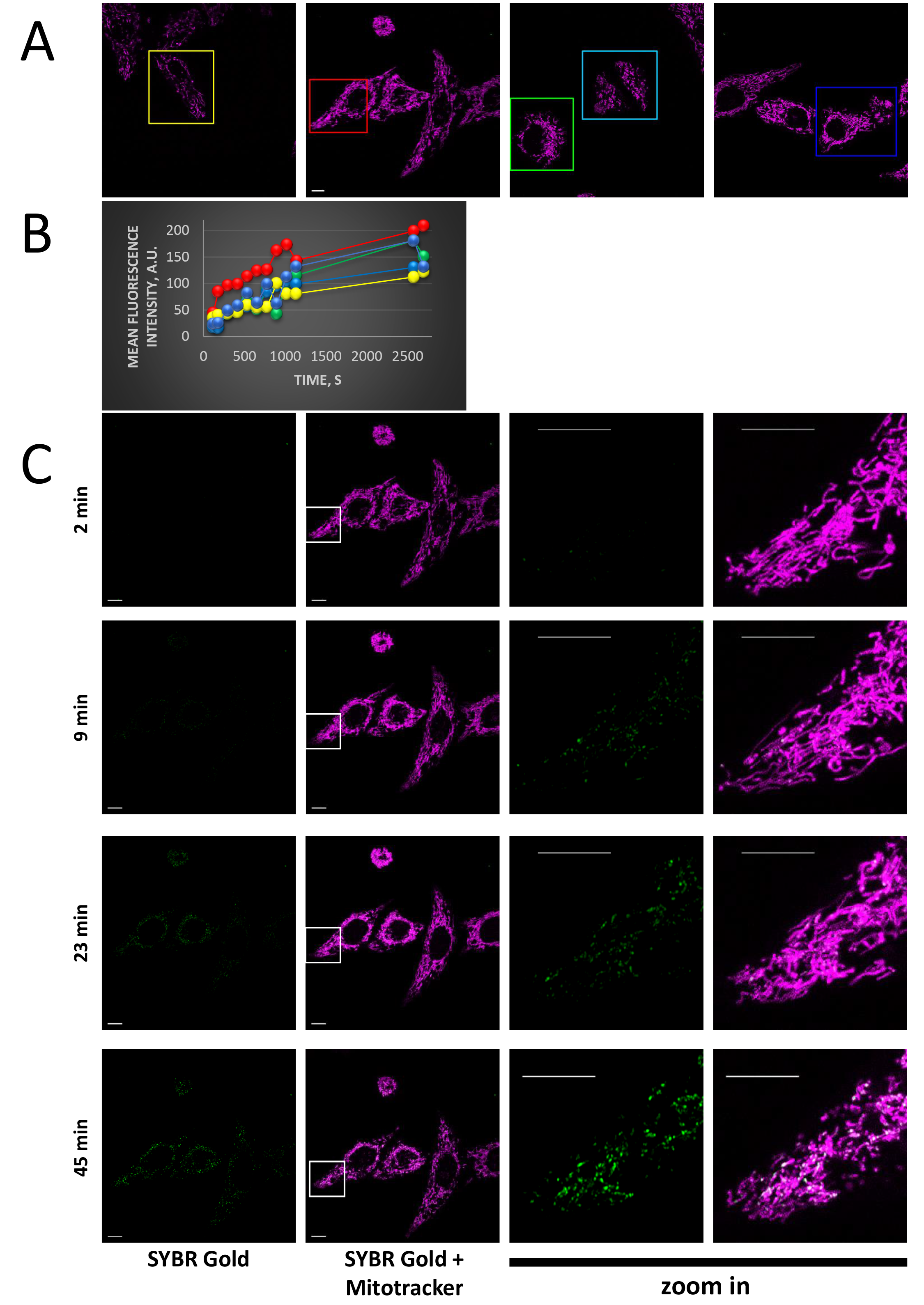

Supplement: S2 Fig — First, live HeLa cells were labelled with Mitotracker CMXRos Red and washed; then SYBR Gold (final dilution 1:10000 in DMEM) was added to the cells and time lapse acquisition has been started. LSM880 microscope, 63x 1.4 Oil objective, sequential acquisition. Z-stacks were acquired at each time point; maximum intensity projections are shown. A. Representative fields of view showing the regions of interest where SYBR Gold fluorescence was measured (colored rectangles). B. Mean intensities of SYBR Gold fluorescence over time in the regions of interest shown on S2A Fig; curve colors correspond to the rectangles on S2A Fig. C. A field of view at several time points during incubation with SYBR Gold. A square region is shown (marked with white line) with higher magnification in the right column. (TIF) [file pone.0203956.s004.tif]

Mitotracker Deep Red

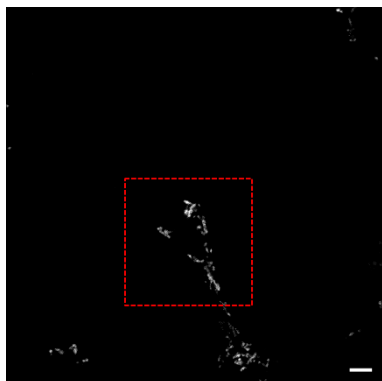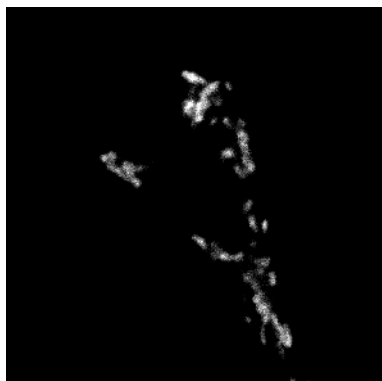

SYBR Gold

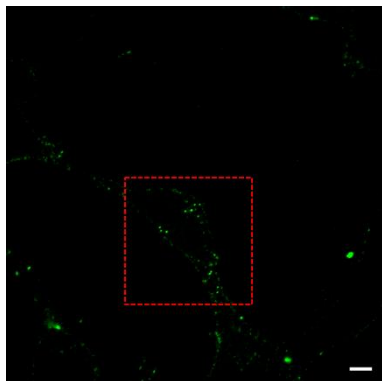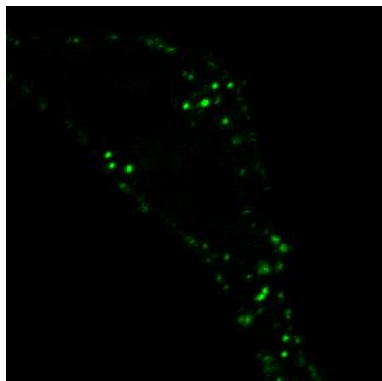

TFAM

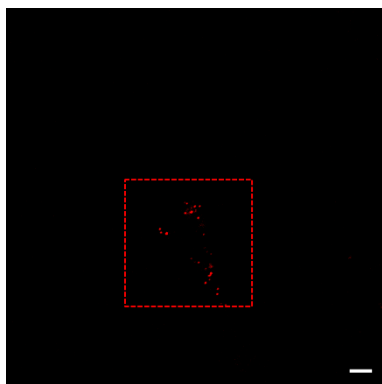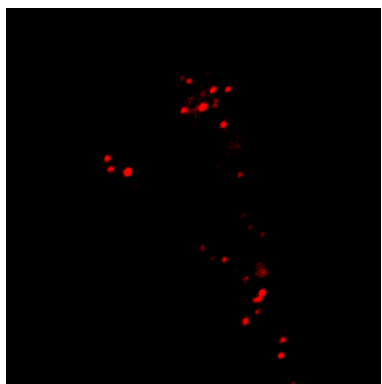

Merge

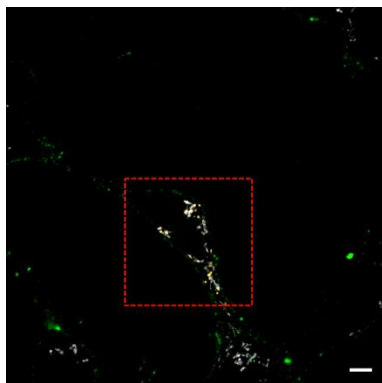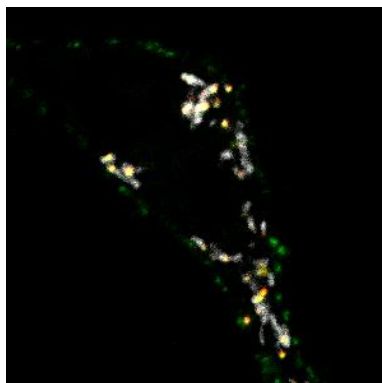

zoom in

Supplement: S3 Fig — Live HEK-T cells; LSM780 confocal microscope, 63x/1.4 Oil objective; 3D image stacks were acquired and used for co-localization analysis. Single optical slice is shown; scale bar 10 μm. Signals in Mitotracker Deep Red (white), TFAM-mEos2 (red) and SYBR Gold (green) channels were acquired sequentially, with switching channels every scanned line. 18 fields of view from two independent transfection experiments were acquired. A representative field of view is shown. Red dashed squares on the left panels mark the region of interest which is shown at higher magnification in the right panels. (PDF) [file pone.0203956.s005.pdf]

Mitotracker Deep Red

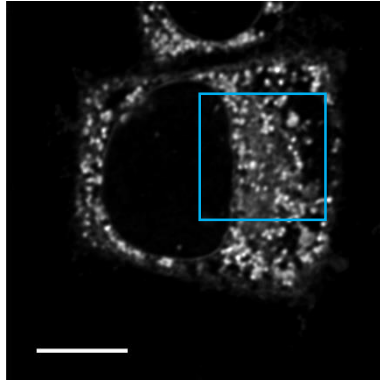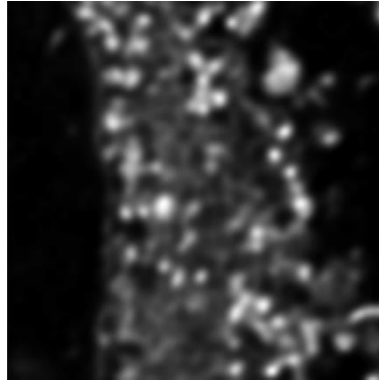

SYBR Gold

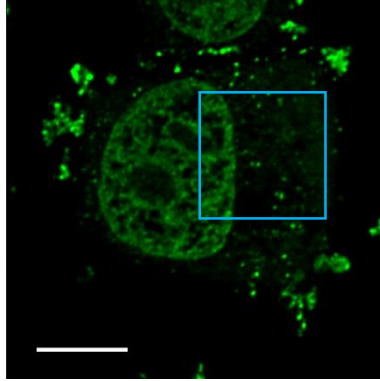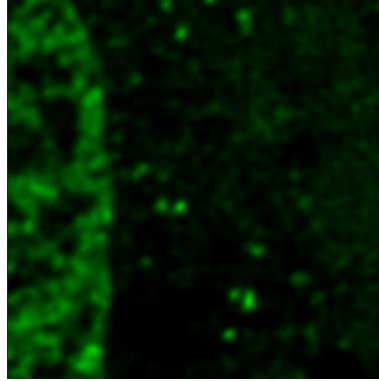

TFAM antibody

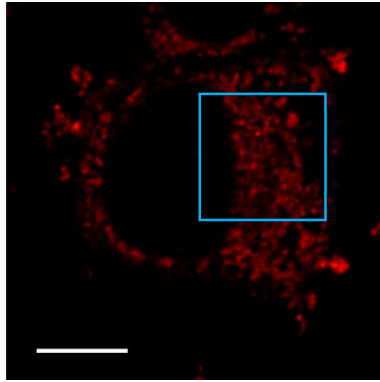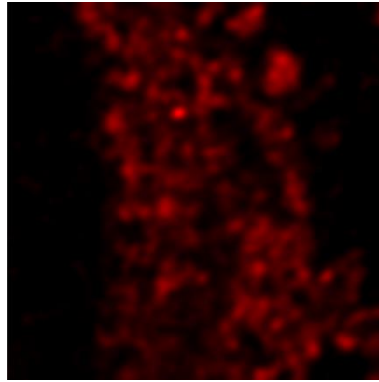

Merge TFAM+SYBR Gold

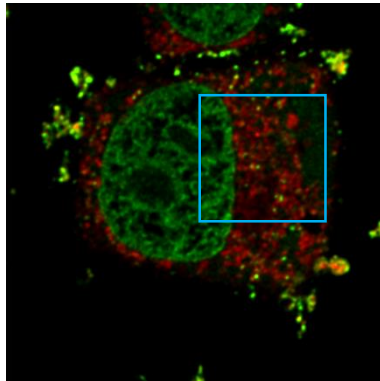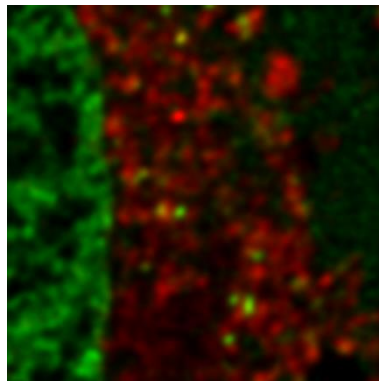

zoom in

Supplement: S4 Fig — Live HeLa cells were transfected with anti-TFAM antibody conjugated to PF555 dye and then stained for 30 min. with SYBR Gold (final dilution 1:10000) and Mitotracker Deep Red™ (final concentration 250 nM). Images (z-stacks) of live cells were acquired on Zeiss LSM780 microscope with 63x/1.4 Oil objective; channels were acquired sequentially; detection ranges were adjusted to minimize spectral bleed-through: (500–550 nm for SYBR Gold, 565–598 nm for PF555 and 645–700 nm for Mitotracker Deep Red. Deconvolution of datasets was performed. Single optical slice from a representative field of view is shown; scale bar 10 μm. Cyan squares on the left panels mark the region of interest which is shown at a higher magnification in the right panels. (PDF) [file pone.0203956.s006.pdf]

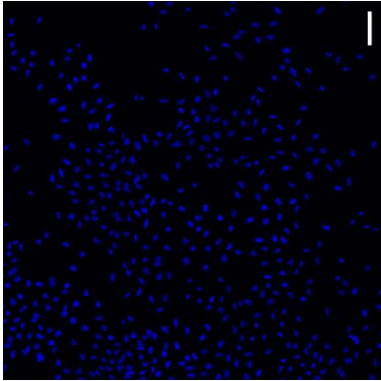

Hoechst 33342

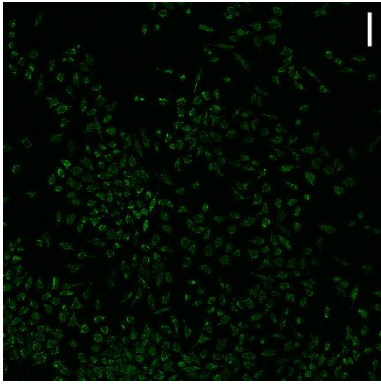

SYBR Gold

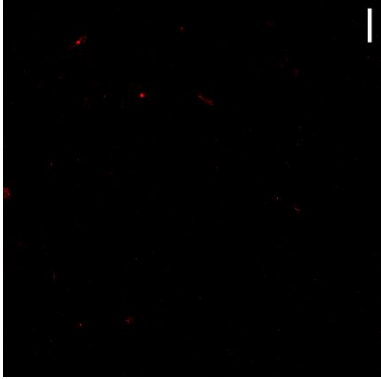

Propidium Iodide

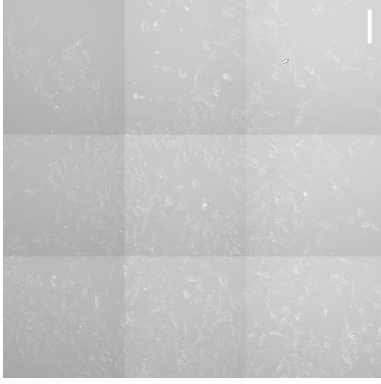

Transmitted light

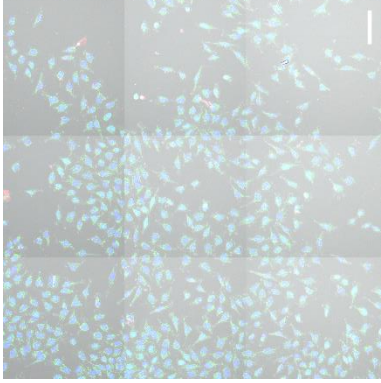

Merge

Supplement: S5 Fig — A representative 1.19×1.19 mm field of view used for calculation of the portion of dead cells (Table 1). Maximum intensity projection of a z-stack is shown. Scale bar 100 μm. (PDF) [file pone.0203956.s007.pdf]

A

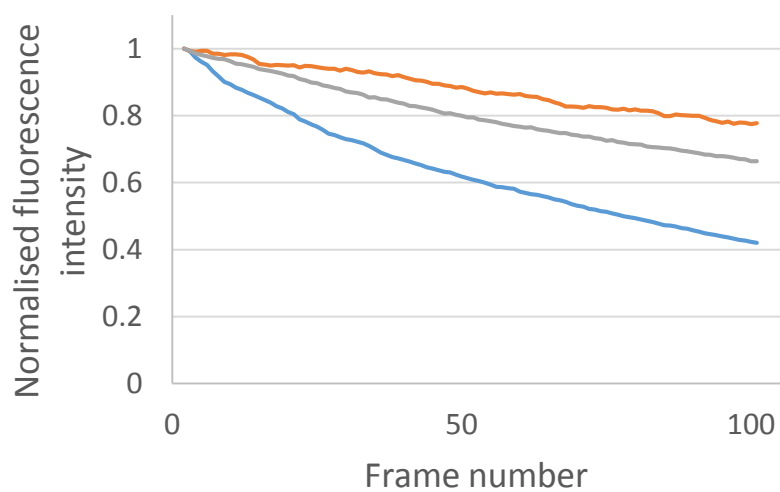

B

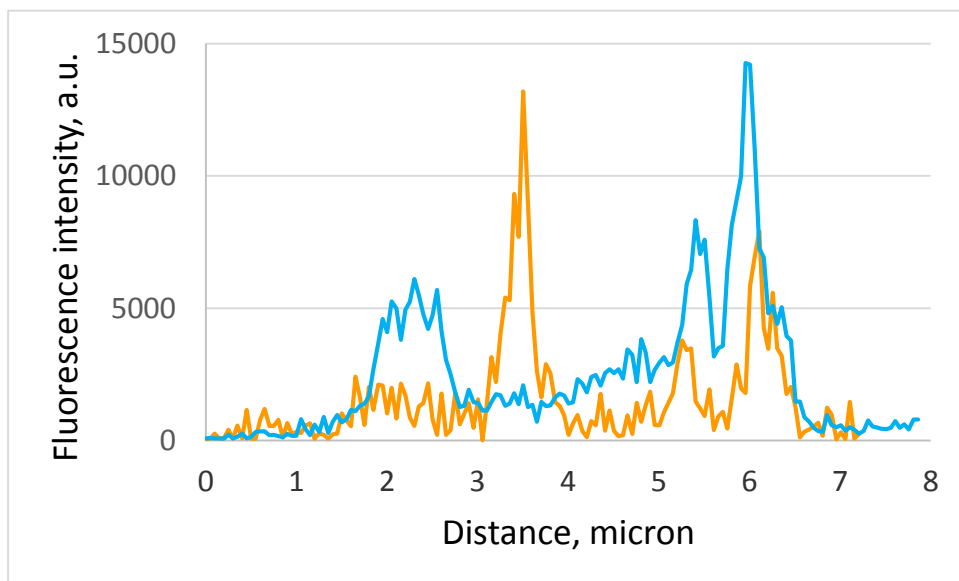

Supplement: S6 Fig — A. Time lapse series of live HeLa cells labelled with SYBR Gold. SIM settings are described in Materials and Methods section. Briefly, frame time 1.8 s, 488 nm laser, 1% AOTF (corresponding to 13.5 microW, 0.54 mW/mm2). Confocal time series were acquired for the same field of view (50 by 50 μm), the same pixel size (50 nm) and same frame time (1.8 s) as for SIM images. Confocal imaging was performed under two settings: 1) blue, the same illumination power as for SIM (13.5 microW); 2) orange, illumination power reduced to 1.6 microW. B. Intensity profiles across nucleoids on confocal images acquired with 13.5 microW (blue) and 1.6 microW (orange). (PDF) [file pone.0203956.s008.pdf]
