# Supplementary material for: Correction: SYBR Gold dye enables preferential labelling of mitochondrial nucleoids and their time-lapse imaging by structured illumination microscopy
Source: PLoS One. 2025 Apr 1;20(4):e0321610. doi: 10.1371/journal.pone.0321610 (PMC11960898; doi:10.1371/journal.pone.0321610)
Supplement: S2 File — (PDF) [file pone.0321610.s002.pdf]

PLOS ONE

RESEARCH ARTICLE

SYBR Gold dye enables preferential labelling of mitochondrial nucleoids and their time-lapse imaging by structured illumination microscopy

Visnja Jevtic, Petra Kindle, Sergiy V. Avilov*

Imaging Facility, Max Planck Institute of Immunobiology and Epigenetics, Freiburg, Germany

* avilov@ie-freiburg.mpg.de

Check for updates

OPEN ACCESS

Citation: Jevtic V, Kindle P, Avilov SV (2018) SYBR Gold dye enables preferential labelling of mitochondrial nucleoids and their time-lapse imaging by structured illumination microscopy. PLoS ONE 13(9): e0203956. https://doi.org/10.1371/journal.pone.0203956

Editor: Vadim E. Degtyar, University of California Berkeley, UNITED STATES

Received: March 16, 2018

Accepted: August 30, 2018

Published: September 18, 2018

Copyright: © 2018 Jevtic et al. This is an open access article distributed under the terms of the Creative Commons Attribution License, which permits unrestricted use, distribution, and reproduction in any medium, provided the original author and source are credited.

Data Availability Statement: All relevant data are within the paper and its Supporting Information files.

Funding: This work was supported by Max Planck Society. The funder had no role in study design, data collection and analysis, decision to publish, or preparation of the manuscript

Competing interests: The authors have declared that no competing interests exist.

Abstract

Mitochondrial DNA molecules coated with proteins form compact particles called mitochondrial nucleoids. They are redistributed within mitochondrial network undergoing morphological changes. The straightforward technique to characterize nucleoids' motions is fluorescence microscopy. Mitochondrial nucleoids are commonly labelled with fluorescent protein tags, which is not always feasible and was reported to cause artifacts. Organic DNA-binding dyes are free of these drawbacks, but they lack specificity to mitochondrial DNA. Here, considering physico-chemical properties of such dyes, we achieved preferential live-cell labelling of mitochondrial nucleoids by a nucleic acid staining dye SYBR Gold. It enabled time-lapse imaging of mitochondrial nucleoids by structured illumination microscopy and quantification of their motions.

Introduction

Genetic material of mitochondria consists of a circular 16.5 kbp DNA molecule, which encodes tRNAs, 22 rRNAs, and 13 polypeptides needed for mitochondrial oxidative phosphorylation complexes. Mitochondria communicate with the rest of the cell in both directions: mitochondrial dysfunction alters expression of nuclear signaling factors (e.g. [1]); on the other hand, nuclear signaling factors affect mitochondrial function: to give few examples, transcriptional co-activator peroxisome proliferator-activated receptor gamma co-activator-1 alpha (PGC-1α) is well-known to control mitochondrial transcription and mitochondrial biogenesis [2]; recently, it was shown that MOF (responsible for acetylation of lysine 16 of histone H4 which is important nuclear transcription activation) binds mtDNA and also affect mitochondrial transcription [3]. mtDNA in complex with mitochondrial transcription factor A (TFAM) and several other proteins form compact structures called nucleoids [4] [5–8]. Mitochondrial network undergoes morphological remodeling, fission/fusion etc, depending on cell cycle phase, stress and other factors (reviewed e.g. in [9]). These morphology rearrangements are accompanied by mitochondrial nucleoids redistributions and motions [10] [11–13]. In addition, mitochondrial nucleoids motions may play a role in diseases: e.g. extrusion of mtDNA

out from neutrophils was reported to be an important step in systemic lupus erythematosus [14]. Light microscopy is the straightforward tool to study organelles in living cells. Regular wide-field microscopy have been used to quantitatively characterize motions of mitochondrial nucleoids [12], within the limits of the instrumentation of that time (2003): at temporal resolution of 1 frame per 15 s and the spatial resolution of ~200 nm, larger than mitochondrial nucleoid size~100 nm [15–17] [18]. "Super-resolution" microscopy developed over last decades, namely STED (stimulated emission depletion) [19] and SMLM (single molecule localization microscopy) [20, 21], as well as electron microscopy, permitted significant progress in characterizing nucleoids structure in fixed samples (e.g. [17, 22] [15, 23]). However, SMLM techniques are rather slow, which limits their suitability to track motions of subcellular structures. To date, few super-resolution observations of mitochondrial nucleoids in live cells were reported, for instance: 1) mtDNA was visualized by dSTORM (direct stochastic optical reconstruction microscopy) together with other DNAs [24], but time lapse series were not acquired; 2) live STED imaging revealed fine sub-mitochondrial structures, whose positions correlated with mtDNA (nucleoids) positions [25]. Although STED is much faster than SMLM (the former can achieve frame rate faster than 1 fps), both STED and SMLM require high illumination doses, which may cause severe phototoxic effects on living cells (e.g. [26]). Thus, non-perturbing time lapse imaging of mitochondrial nucleoids at super-resolution is a current methodological challenge. Here, we have chosen SIM (structured Illumination microscopy) which enables to double the resolution of wide-field images in three dimensions [27]. In contrast to STED and SMLM techniques, SIM does not require high illumination power density [28] [29]. Moreover, SIM is not restricted neither to particular fluorophores nor to imaging buffer composition, while multi-color three-dimensional imaging can be easily implemented [28].

Specific labelling of mitochondrial nucleoids in live cells is not straightforward. The "default" live cell-compatible strategy is fluorescent tagging of a protein associated with mitochondrial DNA, e.g. TFAM [22]. However, this strategy needs overexpression of the tagged construct, which is not feasible in many cases, namely for difficult-to-transfect cell lines and for primary cells. Moreover, overexpression of a fluorescent protein-tagged TFAM caused significant increase of nucleoid number per cell [30], thus producing a serious artifact. Direct DNA labelling with organic dyes has advantages over FP-based strategy. First, nucleic acid-binding dyes can be added to any cell type and at any time point of experiment, without experimental constrains of transfection or stable expression. Second, many nucleic acid-binding dyes, e.g. dyes of TOTO™ and SYTO™ families https://www.thermofisher.com/de/de/home/references/molecular-probes-the-handbook/nucleic-acid-detection-and-genomics-technology/nucleic-acid-stains.html#datatable), have excellent photophysical properties (extinction coefficient and quantum yield), well superior to these parameters of most of FPs (fluorescent proteins) [31]; moreover, no bright FP exist in violet-blue and far-red regions of visible spectrum (http://nic.ucsf.edu/dokuwiki/doku.php?id=fluorescent_proteins). Third, FPs are in general less photo-stable than organic dyes [32]. Finally, organic dyes are much smaller than fluorescent proteins and antibodies, enabling a higher labelling density and more precise localization of the labelled structures (insufficient labelling density may limit the resolution). Several DNA-binding dyes were used for live cell imaging of mitochondrial nucleoids by diffraction-limited microscopy, e.g. DAPI [33], SYBR Green [34], Vybrant DyeCycle [35] [36] and picoGreen [37–39] [24]. However DNA-binding dyes have a substantial drawback for nucleoid labelling: they stain all DNAs of the cell, so most of cellular fluorescence originates from abundant nuclear DNA; moreover, bright fluorescence of nuclear DNA produces stray signal outside the nucleus. Preferential labelling of mitochondrial DNA by an organic dye would be more advantageous, but it has not been reported yet to our best knowledge. To achieve this, rational selection of such a dye was necessary. A range of organic dyes, such as rhodamine 123 and TMRE

(Tetramethylrhodamine Ethyl Ester), are used to stain live mitochondria (e.g. [40]). They are known to accumulate in the live organelles preserving their negative membrane potential, thanks to the following properties of the dye: 1) delocalized positive charge which attracts the molecule to mitochondrial matrix, and 2) lipophilicity, which permits penetration across cellular membranes. The optimal dye for specific labelling of mitochondrial nucleoids should have these properties, in combination with high affinity to DNA and strong enhancement of fluorescence upon DNA binding. Considering these parameters, cyanines constitute a promising group of dyes: on the one hand, cyanines tend to be accumulated in polarized mitochondria (for instance, Cy3 and Cy5 conjugates showed enhanced retention in the organelle [41]); on the other hand, certain cyanines are cell-permeant and bind nucleic acids with high affinity. For instance, a cyanine dye picoGreen stains DNA in live cells and has been used to visualize mitochondrial nucleoids, however, nuclei have been abundantly stained [37–39] [24]. Here, we explored a positively charged unsymmetric cyanine dye SYBR Gold™ (US Patent US5658751), the most sensitive nucleic acid stain *in vitro* [42]. We achieved targeting to mitochondrial nucleoids in live cells by SYBR Gold and demonstrate its suitability for time-lapse SIM in live cells and for quantification of mitochondrial nucleoid motions.

Materials and methods

Reagents and plasmids

Hoechst 33342 (bisbenzimide ethoxide trihydrochloride), acridine orange (3-N,3-N,6-N,6-N-tetramethylacridine-3,6-diamine), Mitotracker CMXRos Red™, Mitotracker Deep Red™, picoGreen™, Propidium Iodide, and SYBR Gold™ were purchased from Thermofisher. The plasmids encoding TFAM-mEos2 and mCherry-MAP4 constructs were kindly provided by Dr. Tim Brown (HHMI, Ashburn, VA, USA) and Dr. Jan Ellenberg (EMBL, Heidelberg, Germany), respectively, and described elsewhere [22], [43].

Cells and labelling

HeLa, Vero and A549 (human lung carcinoma) cells were kindly provided by Dr. Asifa Akhtar (Max Planck Institute, Freiburg, Germany), Dr. Jan Ellenberg (EMBL, Heidelberg, Germany), and Dr. Nadia Naffakh (*Institut Pasteur*, Paris, France). In all experiments, cells were cultured in high glucose DMEM supplemented with 10% FBS, glutamine, penicillin/streptomycin and pyruvate (all from Gibco/Thermofisher). Labelling was performed on live adherent cells at 37˚C in the darkness under 5% CO_2 atmosphere. Stock DMSO solutions of SYBR Gold™, picoGreen™, propidium iodide, Hoechst 33342 and, if appropriate, Mitotracker Deep Red™ or Mitotracker CMXRos Red™ were diluted to needed concentration in phenol red-free DMEM supplemented with 10% fetal bovine serum, glutamine, penicillin/streptomycin and pyruvate. The following final concentrations of the dyes were used for the labelling: 0.5 microG/ml acridine orange; 0.1 microG/ml Hoechst 33342; 10 microM propidium iodide, various concentrations of SYBR Gold and picoGreen (as indicated on the figures); 500 nM Mitotracker Deep Red and Mitotracker CMXRos Red. After incubation with the dyes, the cells were washed with the same medium. For live-cell immunolabelling of TFAM, the rabbit polyclonal anti-TFAM antibody (PA523776, Life Technologies) was covalently attached to PF555 organic dye (PromoKine, Germany) with PromoFluor Protein & Antibody Labelling Kit (PromoKine, Germany). Antibody transfection to HeLa cells was performed with Pierce™ Protein Transfection Reagent (ThermoFisher) according to manufacturer's manual. Adherent cells were incubated for 4 hours with antibody/transfection reagent mixture diluted in OptiMEM medium without serum, then washed 2 times with complete DMEM and then stained with SYBR Gold and Mitotracker Deep Red as described above.

Microscopy samples

All micrographs were acquired on live (or fixed for S4 Fig) cells cultured in glass bottom Ibidi Microslides™ or glass bottom Ibidi 35-mm dishes (Ibidi, Germany), in high glucose phenol red-free DMEM (Gibco/Thermofisher) supplemented with 10% FBS, glutamine, penicillin/streptomycin and pyruvate.

Diffraction-limited microscopy

Adherent cells were imaged with AxioObserver Z spinning disk microscope equipped with Evolve EM-CCD camera (Photometrics), LSM880 Airyscan Fast confocal microscope, and LSM780 confocal microscope, (all from Carl Zeiss, Germany), with 63x/1.4 oil objectives using standard filter combinations for green, red and far red fluorescence to acquire SYBR Gold and SYBR Green signals (green), Mitotracker CMXRos Red and PI (red) and Mitotracker Deep Red signal (far red), except specially described cases. Signals in different color channels were acquired sequentially.

Quantification of SYBR Gold and picoGreen localization upon labelling under various conditions

1.2×1.2 mm fields of view (single confocal slices) were imaged on LSM880 Airyscan Fast microscope (Carl Zeiss, Germany) with a 20x/Air objective. Hoechst 33342 and SYBR Gold or picoGreen signals were acquired sequentially with 405 nm and 488 nm lasers, respectively, with standard filter sets. Since Hoechst dye had been previously reported to emit green fluorescence under certain conditions {Szczurek, 2014 #224}, we controlled for possible bleed-through in the green channel; no signal was detected in Hoechst33342-stained samples in the absence of picoGreen and SYBR Gold (not shown). Quantification of the green (SYBR Gold or picoGreen) signal in the cytoplasm and in the nucleus was performed by a "pipeline" composed in CellProfiler software [44]: nuclei were identified from Hoechst 33342 signal using automatic thresholding; then the nuclei were used to identify cytoplasm by "Distance N" algorithm; finally, intensity parameters of the green signal were calculated for individual nuclei and cytoplasm compartments, and the ratio of these two values was calculated for each cell.

Co-localization of SYBR Gold with TFAM in live cells

HeLa or HEK-T cells transiently expressing TFAM-mEos2 were co-stained for 30 min. with SYBR Gold (final dilution 1:10000) and Mitotracker Deep Red™ (final concentration 250 nM). Images (z-stacks) of live cells were acquired on LSM780 laser scanning confocal microscope with 63x 1.4 oil immersion objective and standard excitation and emission channel settings. Signals were acquired sequentially; to minimize motion-related shift between channels, "switch channels every line" option was used. Quantification of the nucleoid staining by TFAM and SYBR Gold in 3D datasets was performed according to two strategies. 1. Co-localization analysis using Coloc module of the Imaris 8.1.1 software (Bitplane, Switzerland), with automatic threshold setting. Pearson coefficient was calculated for ROIs representing mitochondria; ROIs were created from the masks comprising mitochondria (Mitotracker Deep Red staining). 2. Nucleoids were detected and counted as "spots" using "Spots" module of Imaris 8.4.1 software, independently in TFAM channel and in SYBR Gold channel. To estimate if all "TFAM-positive" spots are labelled with SYBR Gold as well, the raw datasets were masked with SYBR Gold channel (such that only SYBR Gold-positive volume remained in the datasets), and TFAM detection was performed again within this masked volume. The portion of

SYBR Gold-stained nucleoids was calculated as the ratio of nucleoid number in the “SYBR Gold-masked” 3D image to the nucleoid number in the same 3D image before masking.

Testing SYBR Gold effect on cell viability

On day 1, the adherent cells cultured in 35-mm dishes were incubated with the SYBR Gold at various dilutions; on day 3, cells were stained with PI and Hoechst 33342. Immediately after staining, live cells were washed with phenol red-free DMEM and imaged on LSM880 microscope (Carl Zeiss) with a 20x air objective. Propidium iodide, SYBR Gold and Hoechst signals were acquired sequentially with 561, 488 and 405 nm excitation, respectively, with standard filter sets, in 1.19×1.19 mm fields of view. Then, total number of cells was determined by counting the nuclei in Hoechst 33342 channel, while number of dead cells was determined from counts of PI-stained cells, using a pipeline written in CellProfiler software [44].

Effect of mitochondrial membrane potential on SYBR Gold localization

HeLa or A549 cells were incubated for 15–30 min. with FCCP (carbonyl cyanide-4-(trifluoromethoxy)phenylhydrazone) at indicated concentrations, or with DMSO; then SYBR Gold and Mitotracker CMXRos Red or Mitotracker Deep Red were added to the medium containing FCCP. After 30 min of labelling, the solution of the dyes was replaced with the same medium containing FCCP, and images of live cells were acquired on LSM880 Airyscan Fast confocal microscope with 63x/1.4 oil objective and standard filter sets.

SIM

SIM was performed on Elyra PS1 microscope (Carl Zeiss), with Plan Apochromat 100x/1.46 Oil objective, 1.6x lens in the detection light path and Andor iXon 885 EMCCD camera; image pixel size was 50 nm; SIM raw images were acquired with 3 grid rotations and 5 phases, at 50 ms exposure for each orientation and phase combination. In multi-color images, channels were acquired sequentially using standard single-band filter sets. Z-stacks were acquired with 100 nm intervals. Raw SIM images were processed with Zen Black 2012 software (Carl Zeiss). 2D time lapse series of 50–100 frames were acquired at the speed of 1.8 s per frame, in SYBR Gold channel only. Confocal imaging for comparison with SIM was performed using the LSM light path of Elyra PS1 microscope, which enabled acquisition of the same fields of view in confocal and SIM modes, using Plan Apochromat 100x /1.46 Oil objective, with pixel size matched to that of SIM images.

Quantification of mitochondrial nucleoids positions

The nucleoids were detected and their positions were determined by “Spots” module of Imaris 8.4.1 software. *Spot-to spot closest distance* plug-in (“Xtension”) of Imaris XT module was used to calculate for each spot the distance (in 3D) to the closest neighbor; then distributions of these distances (pooled data from multiple 3D images) were plotted.

Mitochondrial nucleoids tracking

Mitochondrial nucleoids tracking on SIM 2D time lapse series (SYBR Gold channel) was done by Lineage module of Imaris 8.4.1 software (Bitplane, Switzerland) with “Autoregressive Motion” algorithm, with maximum gap of 1 frame and with longest distance between nucleoid positions in the consecutive frames set to 0.45 μm; tracks shorter than 4.5 s were discarded. Mitochondria contours were not imaged during tracking, and their positions were not used as “constrains” for software to build the tracks. Tracks parameters (mean speed, maximal instant

speed, etc) were calculated for each track individually. Further, the values of each parameter for individual tracks under each condition (control or nocodazole-treated cells) were combined in a worksheet (e.g., maximum instant speeds for all tracks in control cells), and histograms showing distributions of a parameter were plotted.

Results

SYBR Gold preferentially stains mitochondrial nucleoids at low concentration

First, we evaluated intracellular distribution of picoGreen and SYBR Gold upon incubations at several dilutions of these dyes (Fig 1). At high concentrations (dilutions of commercial stock 1:1000–1:2000), SYBR Gold and picoGreen mostly labelled the nuclei and showed a punctate staining in the cytoplasm, in agreement with published data on the latter dye [24]. Importantly, SYBR Gold fluorescence intensity was much higher than that of picoGreen upon labelling and imaging under identical conditions. Considering that, we tried to label live cells at lower concentrations, expecting that specific attraction of a positively charged dye to active mitochondria will be more pronounced under these conditions. Indeed, we observed that upon short (1 hr or less) incubation at 1:10000 dilution, SYBR Gold stained nuclei only weakly, while bright green dotted pattern appeared in mitochondria (Fig 1); the ratio of integrated cytoplasmic signal intensity to integrated nuclear signal intensity was 1.06 (Fig 2), which corresponds to highly preferential staining of mitochondrial nucleoids, considering their small volume and small total mtDNA content, in comparison to that of the nucleus. In addition, at 1:10000 dilution, the nucleoids are much brighter than the nuclei (brightness per pixel), which will permit one to filter nuclear signal out during image analysis, when only nucleoids signal is of interest. PicoGreen at 1:10000 dilution showed a staining pattern similar to that of SYBR Gold; however fluorescence was much dimmer, hampering reliable signal quantification. (Fig 1). We tried to stain live cells at a wider SYBR Gold concentration range (S1 Fig) with simultaneous co-staining of mitochondria with Mitotracker CMXRos Red. At 1:500 and 1:1000 dilutions, SYBR Gold localized both in the nuclei and in mitochondria, while at 1:10000 and 1:50000 dilutions, most of staining appeared in mitochondria. Moreover, after labelling at 1:500 and 1:1000 SYBR Gold dilutions, mitochondria appeared more fragmented than after labelling at 1:10000 and 1:50000 dilutions, suggesting possible perturbation of the cells at the higher concentrations. Direct observations of the cells incubated with SYBR Gold solution (S2B Fig) showed that accumulation of the dye nearly reached a plateau after ~20 min.

Further, we evaluated stability of SYBR Gold localization over time (Fig 3). In the cells cultured for 2 days after 1-hr long labelling, SYBR Gold localization was very similar to that immediately after labelling (Fig 1), suggesting that SYBR Gold does not redistribute between compartments spontaneously, and thus can be suitable for long-term nucleoids tracking. Interestingly, when cells were cultured for 2 days in the presence of 1:10000 diluted SYBR Gold, then the dye localization was similar to that observed upon short incubation high concentrations: both nuclei and mitochondrial nucleoids were brightly stained.

To confirm that the SYBR Gold-positive spots are mitochondrial nucleoids, we labelled with SYBR Gold (diluted 1:10000) HeLa cells transiently expressing TFAM-mEos2 fusion protein, a well-known mitochondrial nucleoid marker [8, 17]. In addition, it was important to estimate the portion of nucleoidswhich are SYBR Gold-positive, because a similar dye picoGreen had been reported to preferentially stain relaxed DNA and had been hypothesized to "omit" a subpopulation of nucleoids [38]. Here, we observed that SYBR Gold spots in the cytoplasm mostly co-localized with TFAM-Eos2 spots (Fig 4). Further, we calculated the portion of TFAM-positive spots which were also stained by SYBR Gold (see Materials and Methods) and

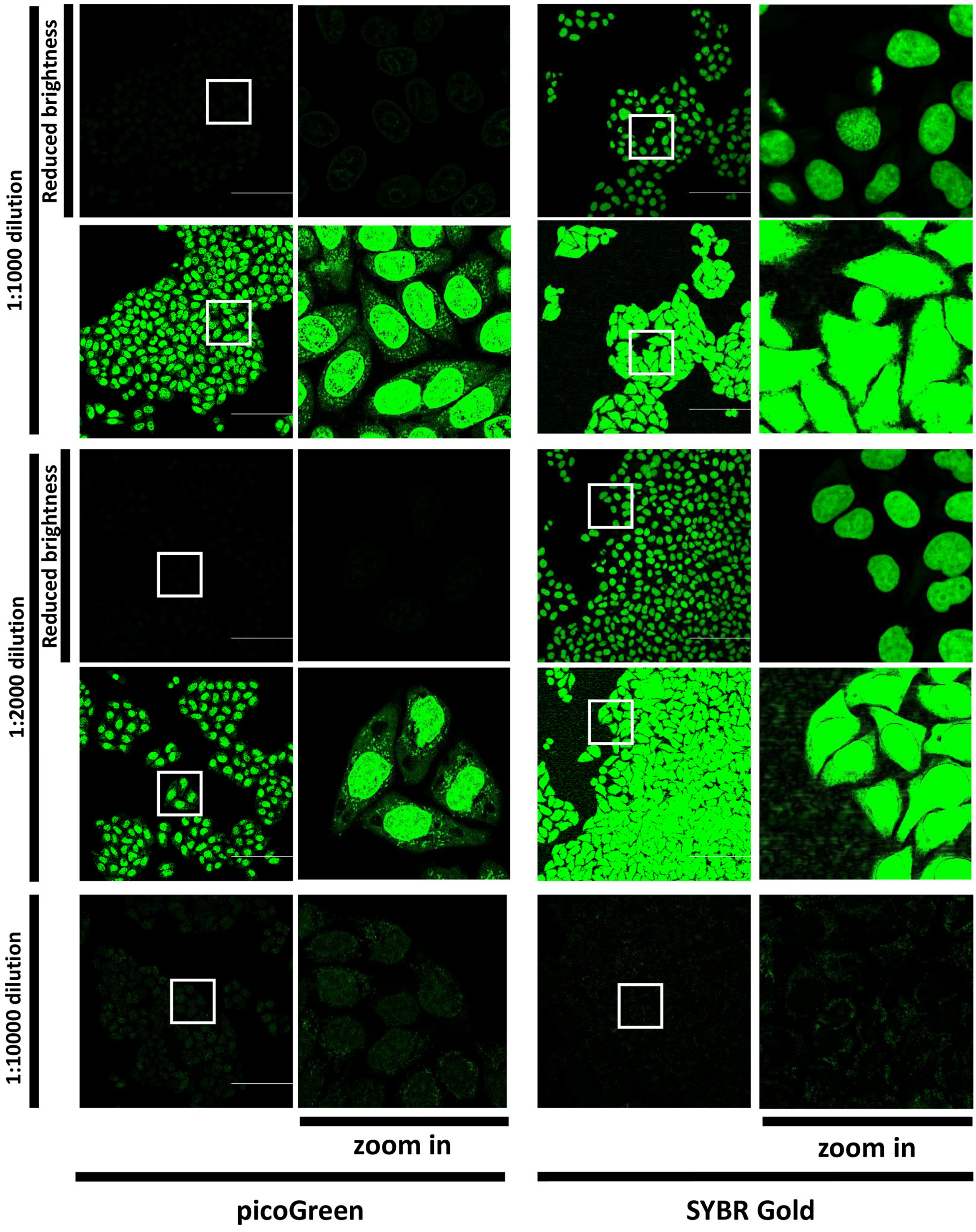
Reduced brightness
1:1000 dilution
Reduced brightness
1:2000 dilution
1:10000 dilution
zoom in
zoom in
picoGreen
SYBR Gold

Fig 1. Comparison of live cell labelling with picoGreen and SYBR Gold at various dilutions. A549 cells were incubated with Hoechst 33342 and picoGreen or SYBR Gold at indicated dilutions and imaged. Representative fields of view of the labeled cells in the "green" channel. LSM880 Airyscan Fast, 20x/Air objective, scale bar 50 μm. Single optical sections. White squares mark the areas shown at a higher zoom to the right of the entire 423×423 μm fields of view. For 1:1000 and 1:2000 dilutions, the same images are shown at two brightness settings: the "default" brightness settings optimized for 1:10000 dilution, and the "reduced brightness" settings adjusted to minimize saturation.

https://doi.org/10.1371/journal.pone.0203956.g001

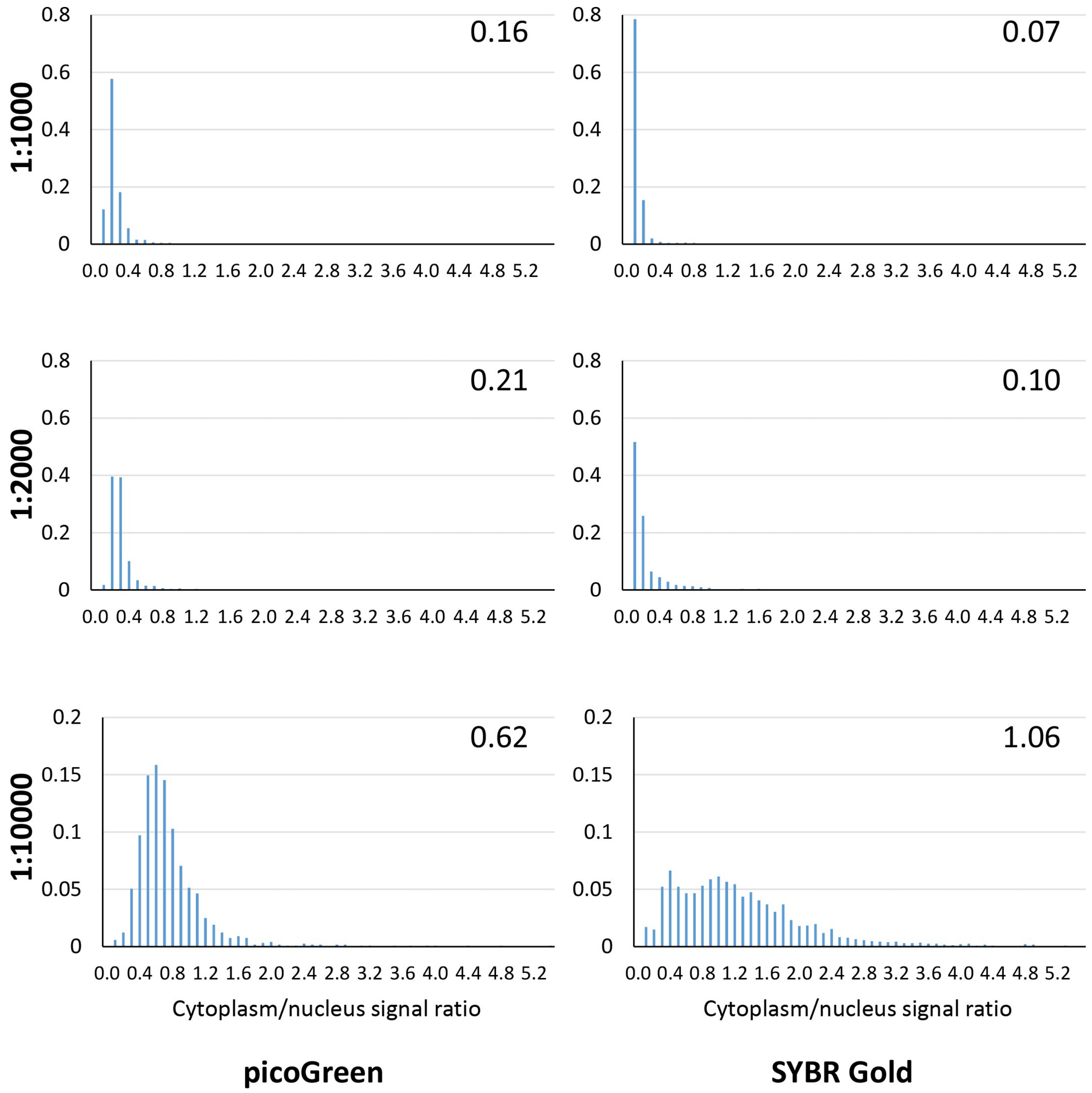

Fig 2. Distribution of the cytoplasmic to nuclear signal ratio in the living cells labelled with picoGreen or SYBR Gold. Nine 423×423 μm fields of view per condition were used for the quantification. The ratio of the integrated cytoplasmic and nuclear signals was calculated for each cell (n = 841. . .2769). Normalized distributions of these ratios are shown. Median value for each distribution is shown in the upper right corner. Note different scale on *y* axis for 1:10000 dilution.

https://doi.org/10.1371/journal.pone.0203956.g002

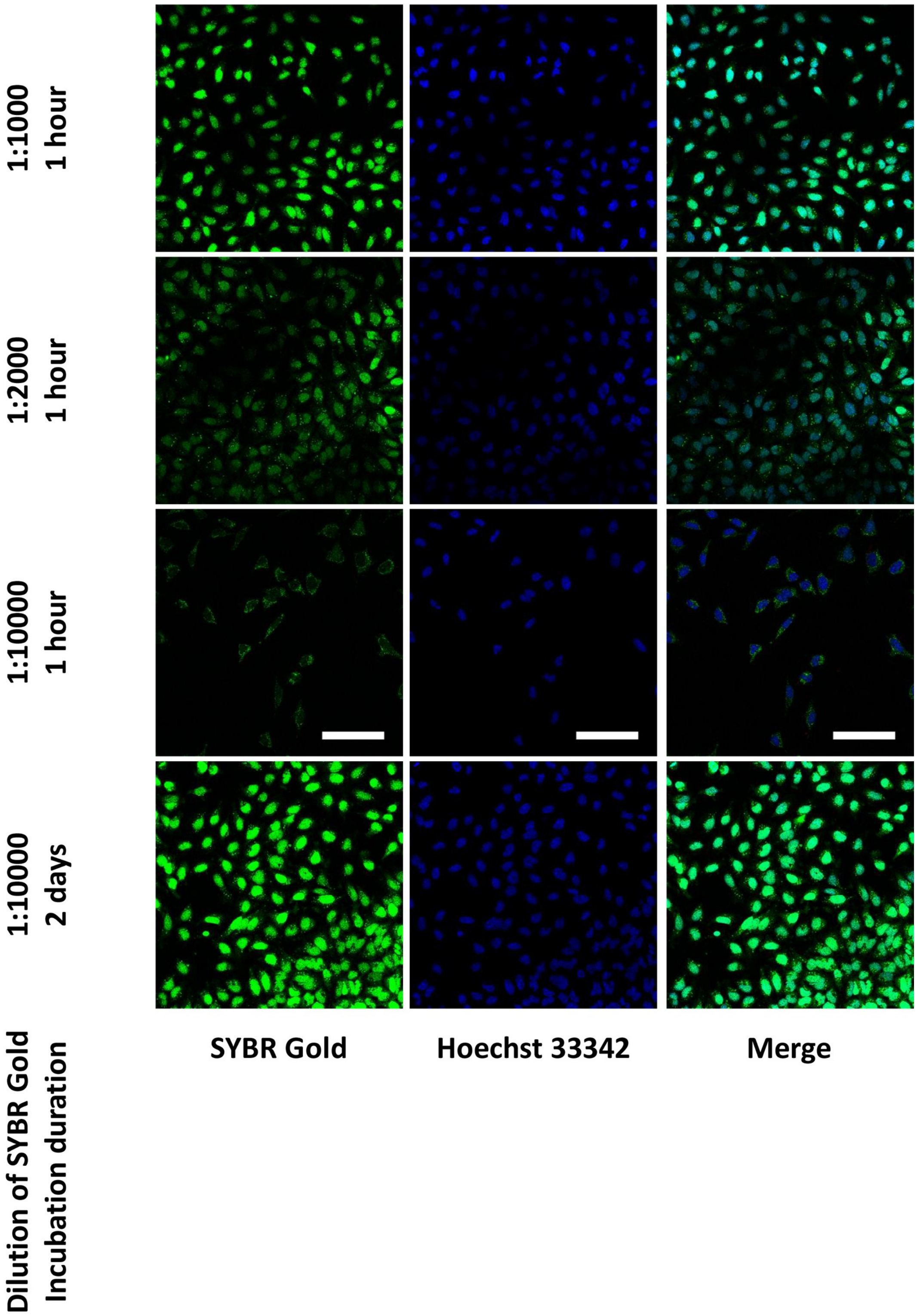

Fig 3. Effect of incubation duration and concentration on SYBR Gold localization in live cells. Indicated dilutions of SYBR Gold in DMEM were added to live HeLa cells in simultaneously; cells were kept with SYBR Gold for 1 hour or for 2 days (as indicated on the Figure). Then, cells were stained with Hoechst 33342 and imaged in parallel (i.e. all samples were imaged 2 days after the start of incubation). LSM880 confocal microscope; 20x/Air objective; scale bar 100 μm. 415×415 μm fields of view are shown. Further details are described in Materials and Methods.

https://doi.org/10.1371/journal.pone.0203956.g003

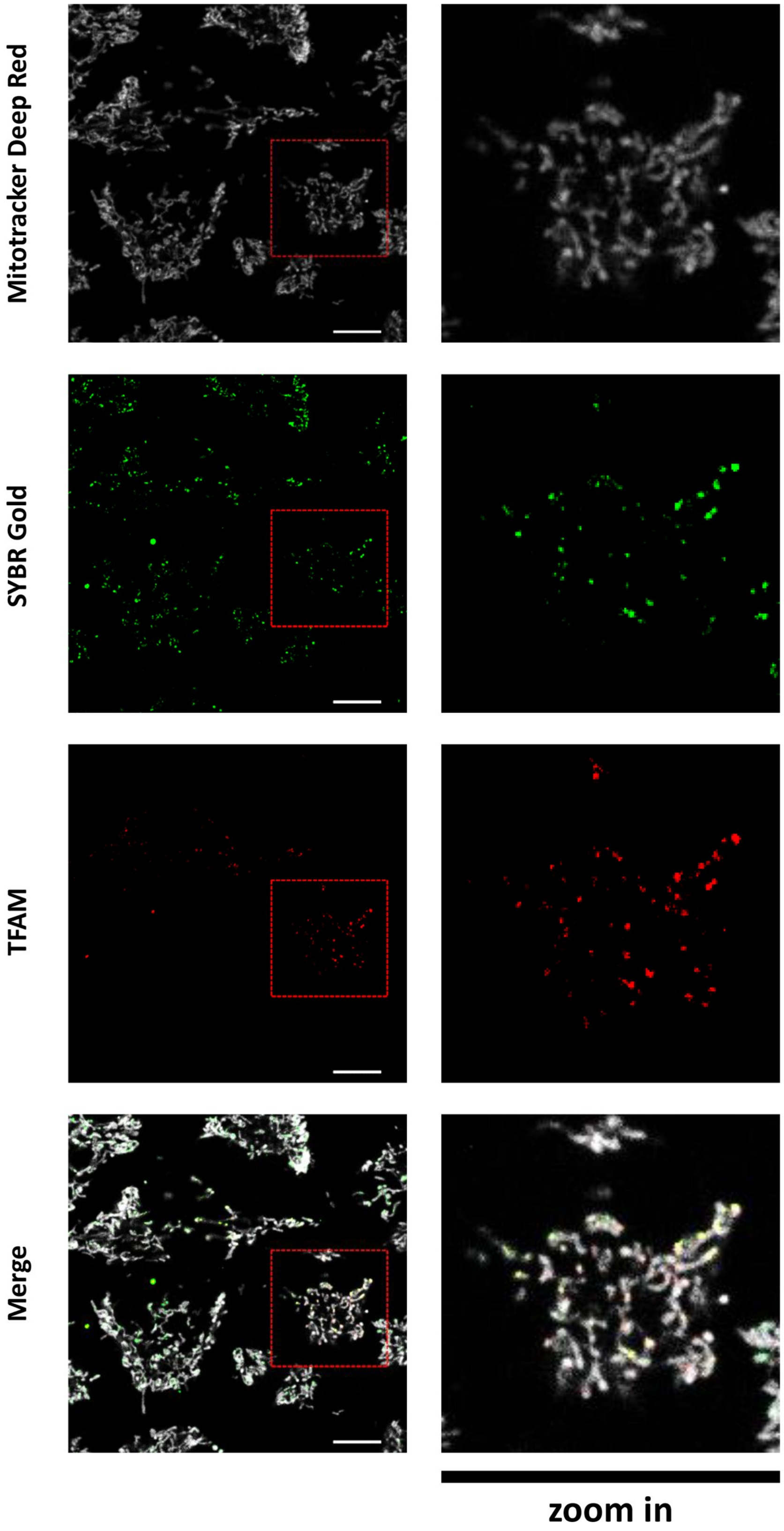

Fig 4. Co-localization of SYBR Gold with TFAM. Live HeLa cells transiently expressing TFAM-mEos2 and stained for 30 min. with SYBR Gold (final dilution 1:10000) and Mitotracker Deep Red™ (final concentration 250 nM). Images

(z-stacks) of live cells were acquired and used for analysisPS1 Elyra, confocal mode, 100x/1.46 Oil objective; single optical slice from a representative field of view is shown; scale bar 10 μm. Red dashed squares on the left panels mark the region of interest which is shown at higher magnification in the right panels.

https://doi.org/10.1371/journal.pone.0203956.g004

found it to be 0.87+/-0.11 (mean+/-SD, n = 9). In HEK-T cells, similar results were obtained: 0.84+/-0.14 (mean+/-SD, n = 18) (S3 Fig). These observations confirmed that most of mitochondrial nucleoids are stained with SYBR Gold. To obtain an additional proof that SYBR Gold-positive spots in cytoplasm represent mitochondrial nucleoids, we aimed to estimate SYBR Gold co-localization with immunostained endogeneous TFAM. However, we found that SYBR Gold staining is strongly perturbed by permeabilization (see the next chapter); this made standard immunofluorescence protocols incompatible with SYBR Gold staining. To circumvent the problem, we delivered fluorescent anti-TFAM antibody directly to live cells using a protein transfection reagent, and then labelled the cells with SYBR Gold and Mitotracker. We observed that mitochondrial SYBR Gold spots localize within the areas stained by the TFAM antibody (S4 Fig). Mediocre penetration of the TFAM antibody into mitochondria and non-specific staining by this antibody outside mitochondria did not permit us to quantify co-localization of endogeneous TFAM with SYBR Gold. Nevertheless, this observation supports our conclusion (based on robust co-localization data on fluorescent protein-tagged TFAM) that SYBR Gold spots in the cytoplasm are mitochondrial nucleoids.

SYBR Gold localization in cells depends on mitochondrial membrane potential and is perturbed by fixation and permeabilization

Since SYBR Gold molecule has a net positive charge at neutral pH (US Patent US5658751), we speculated that the negative membrane potential of intact "live" mitochondria should contribute to specific accumulation of that dye. To confirm this hypothesis, we studied how SYBR Gold localization is affected by membrane-permeant ionophore FCCP which dissipates mitochondrial membrane potential. We observed that if labelling was performed after incubation with FCCP, then mitochondrial SYBR Gold subpopulation decreased, while its non-mitochondrial subpopulation increased, with most pronounced effect at 10 microM FCCP (Fig 5). This observation shows contribution of the mitochondrial membrane potential to preferential labelling of mitochondrial DNA with this dye. Considering the data shown on Figs 3 and 5, we speculate that electrostatically-driven accumulation of SYBR Gold in mitochondria is relatively fast and prevails at low concentrations (dilution 1:10000) during the first hour of incubation, while at higher concentrations of the dye or after the longer incubation (2 days), SYBR Gold molecules bind all DNA in the cell, which leads to predominant staining of abundant nuclear DNA. Furthermore, fixation (2% PFA) of the cells live-stained with SYBR Gold slightly increased nuclear subpopulation of the dye (Fig 6B). Upon subsequent permeabilization with 0.1% Triton X100 detergent, SYBR Gold dotted pattern disappeared from mitochondria, while dim diffuse staining of the nuclei became predominant (Fig 6C and 6D). If SYBR Gold was added to cells after fixation and permeabilization, then the entire cells were brightly and diffusely stained within few minutes (Fig 6D).

Thus, summarizing the data described above, we identified the labelling conditions which permitted preferential labelling of mitochondrial DNA by SYBR Gold, thanks to its advantageous physico-chemical properties: very high brightness of DNA-bound form, net positive charge and membrane permeability. PicoGreen dye, like SYBR Gold, preferentially binds mtDNA at low concentrations (1:10000), but its fluorescence is much dimmer under identical image acquisition settings, making picoGreen much less suitable for preferential staining of nucleoids. We do not use the term "specific labelling" since a subpopulation of SYBR Gold is

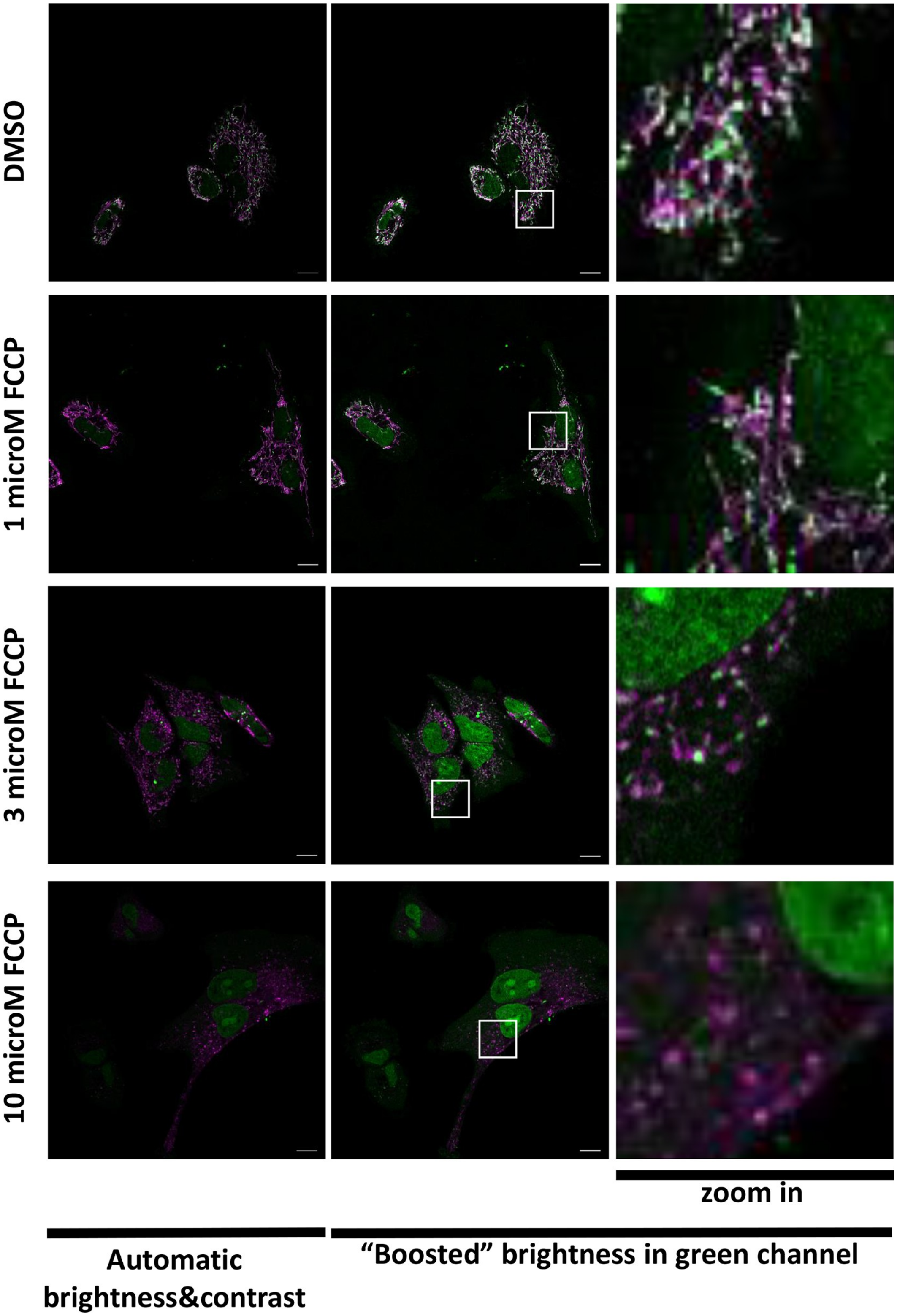

Fig 5. Effect of FCCP on SYBR Gold localization. HeLa cells were pre-incubated with FCCP and then labelled with SYBR Gold (dilution 1:10000) and Mitotracker Deep Red in the presence of FCCP. Merged SYBR Gold (green) and Mitotracker Deep Red (magenta) channels are shown. White squares mark regions of interest which is shown on zoomed images. Individual confocal slices. LSM880; 63x/1.4 Oil objective.

https://doi.org/10.1371/journal.pone.0203956.g005

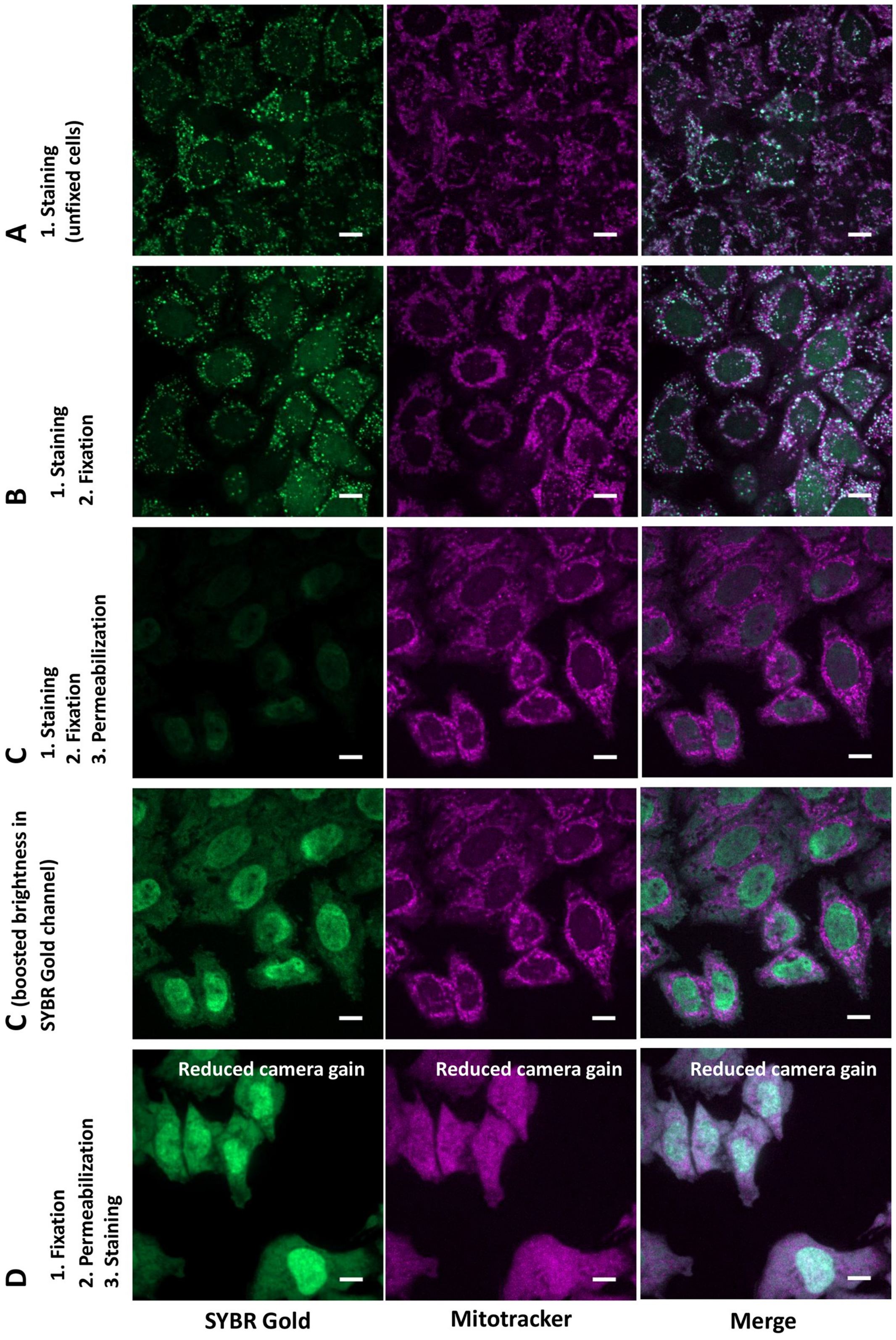

Fig 6. Effect of fixation and permeabilization on SYBR Gold localization in the cells. HeLa cells were stained with SYBR Gold (stock diluted 1:10000) and Mitotracker CMXRos Red (250 nM) for 30 min. Single optical slices acquired on spinning disk microscope; sequential acquisition; scale bar 10 μm. Other conditions are further described: A. Live HeLa cells stained

with SYBR Gold; B. HeLa cells stained alive with SYBR Gold and then fixed with 2% PFA in PBS for 30 min; C. HeLa cells stained alive with SYBR Gold, then fixed with 2% PFA in PBS for 30 min and permeabilized with 0.1% Triton X100 for 15 min; on the lower C panel, the same image is shown, but brightness in green channel is set higher; D. HeLa cells fixed with PFA, then permeabilized with 0.1% Triton X100 for 15 min. and finally stained with SYBR Gold and Mitotracker CMXRos Red. To acquire images shown on panel D, EMCCD gain of the camera for green channel was reduced by factor of 12 in comparison to A-C, to avoid overexposure.

https://doi.org/10.1371/journal.pone.0203956.g006

always present in the nucleus, and since "specificity" in biology frequently refers to "lock-and-key" binding. However our data show that under optimal conditions, the dye preferentially stains mtDNA. This constitutes a significant advantage over "all-DNA" stainings reported so far. Perturbing mitochondrial membrane potential by an ionophore and destruction of membranes by a detergent hamper efficient accumulation of SYBR Gold in the nucleoids. This prevents combining SYBR Gold with standard IF protocols, however it enables one to preferentially label and observe only the nucleoids in "healthy", non-perturbed mitochondria.

Labelling with SYBR Gold does not reduce cell viability

To estimate if SYBR Gold labelling exerts toxicity, we incubated HeLa cells with the dye and 2 days later quantified their viability by dead cell staining with propidium iodide. Both in control and in SYBR Gold-labelled samples (representative field of view is shown on S5 Fig), portion of dead cells was <1% (except labelling at 1:2000 dilution, where the portion was 1.14±0.60%); difference with control was not significant for all tested labelling conditions (Table 1). This indicates that SYBR labelling does not affect cell viability and is suitable for long live cell observations.

SYBR Gold staining is suitable for live cell SIM imaging of mitochondrial nucleoids

Optimal SIM imaging needs oil immersion objectives with high numerical aperture. For live-cell observations, refractive index mismatch between immersion oil and a water-based cell culture medium might compromise the SIM image quality. Therefore, it was necessary to estimate resolution of our set up for the samples in aqueous media using a reference sample. For this purpose, here we imaged 100 nm Tetraspek fluorescence beads under the same cell culture medium and the same microscope settings as used for live mitochondrial nucleoids. We obtained FWHM of 262 nm in confocal mode and 166 nm in SIM mode (Fig 7). Thus, our live SIM set up should resolve closely positioned nucleoids which would not be resolved by confocal microscopy. However, the diameter of nucleoids (90–110 nm, as determined by EM and STED [17]) is close to theoretical resolution limit of SIM microscopy (ca 100 nm), so the latter should not be used to determine real size and shape of mitochondrial nucleoids.

Table 1. Effect of SYBR Gold labelling under various conditions on cell viability.

Sample	Portion of dead cells, %
SYBR Gold dilution 1:10000, 2 days	0.63±0.06
SYBR Gold dilution 1:10000, 1 hr.	0.66±0.25
SYBR Gold dilution 1:2000, 1 hr.	1.14±0.62
SYBR Gold dilution 1:1000, 1 hr.	0.52±0.27
Control (DMSO)	0.79±0.52

Portion of dead cells is calculated independently for each of 2. . .4 biological replicates, and then standard deviation is calculated.

https://doi.org/10.1371/journal.pone.0203956.t001

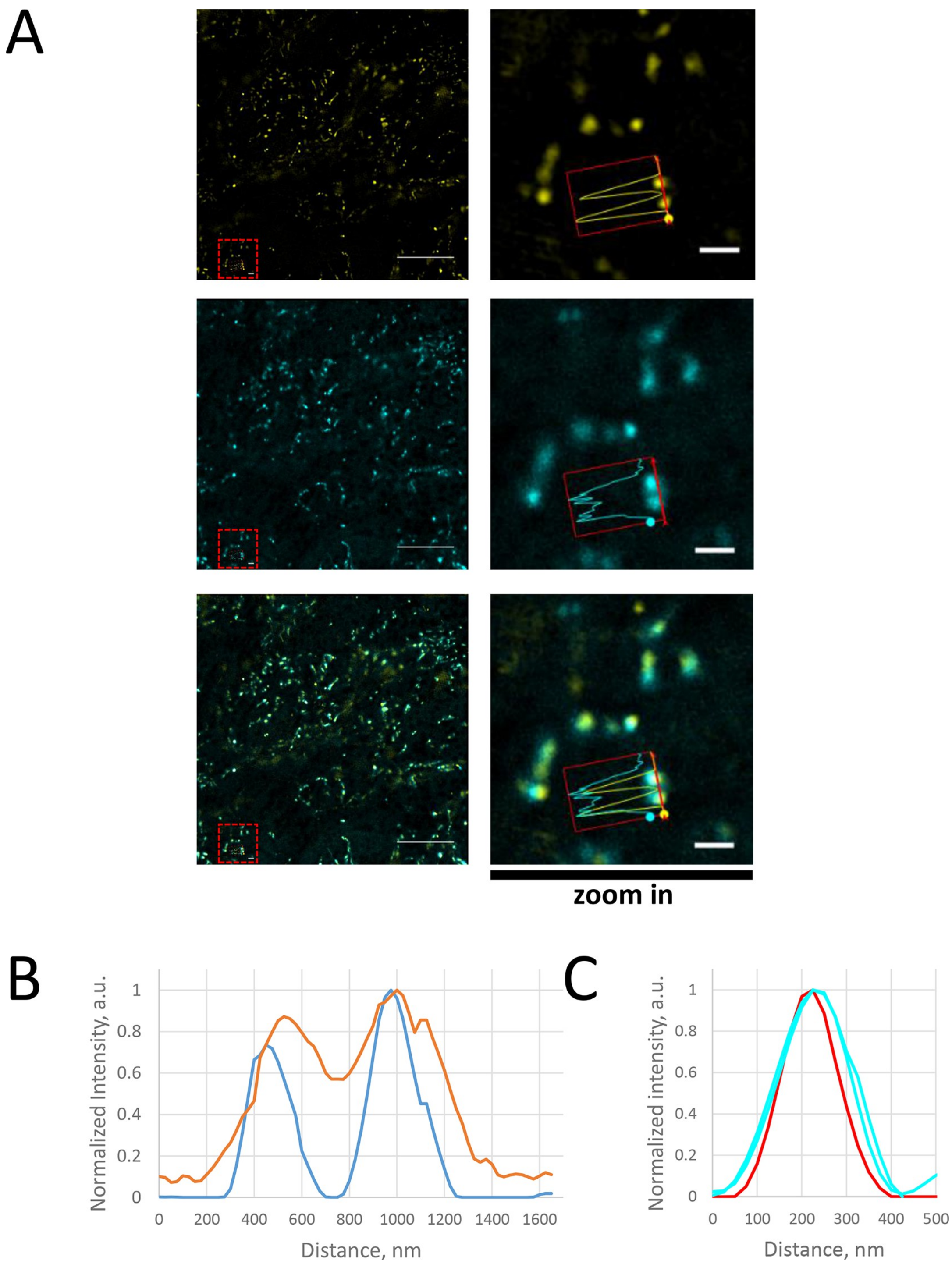

Fig 7. Comparison of confocal (cyan) and SIM (yellow) imaging of live cells labelled with SYBR Gold. A. Representative images (single optical slices). The same objective 100x 1.46 Oil TIRF and the same sampling (50 nm/pixel) is used for both LSM and SIM images. Left column shows entire field of view; Scale bar 10 μm; ROI is marked with red dashed line; ROI is shown in the right column with higher magnification (scale bar 1.25 μm). B. An intensity profile across two nucleoids (marked as a red arrow on higher magnification panels) in confocal (orange) and SIM (cyan) channels. C. Intensity profiles of fluorescent bead (red) and mitochondrial nucleoids (cyan) in SIM images. Further, we acquired SIM 3D datasets of live cells co-stained with SYBR Gold and Mitotracker Deep Red. Mitochondrial nucleoids appeared as bright spots within mitochondria, frequently as rows with equal distances between neighbors (Fig 8A). Distribution of distances between neighbor nucleoids showed a single maximum at 450 nm approx. (Fig 8B). This agrees with the values of 400–800 nm previously reported for fixed mammalian

cells (Fig 1E in [45]). Interestingly, peaks in SYBR Gold channel intensity profiles correspond to local minima on the intensity profiles in Mitotracker Deep Red channel (Fig 8A, bottom panels), suggesting that freely diffusing mitochondrial matrix proteins are excluded from nucleoids; this agrees with previously reported exclusion of a mitochondria marker YFP-tagged cytochrome oxidase from nucleoids [45]. Thus, SIM imaging of SYBR Gold-stained cells enabled us to reproduce in live cells the observations of mitochondrial nucleoids, previously done in fixed cells and with diffraction-limited microscopy.

https://doi.org/10.1371/journal.pone.0203956.g007

Time-lapse SIM

Temporal resolution of SIM is modest, since each processed SIM image requires several raw images acquired with different phases and orientations of the grid. To minimize frame time in SIM, the fluorophore should be as bright as possible. SYBR Gold is a promising dye: in complex with nucleic acids it has quantum yield of ~0.7 [42]. We found that reconstruction of super-resolved images of SYBR Gold-stained nucleoids is possible from raw SIM images acquired at 1% power of 488 nm laser, EM-CCD gain of 50. . .100 and raw image exposure time of 50 ms (yielding final SIM frame time of 1.8 s for 3 grid rotations and 5 phases). We quantified photobleaching caused by image acquisition under these illumination conditions. After acquiring SIM images in 100 time points, signal intensity dropped to 66% of its initial level (S6A Fig). For comparison, time lapse series of the same sample were acquired using LSM light path, with the same frame time, same field of view size and same pixel size. Two illumination power values were used for confocal imaging: 1) 13.5 microW, equal to the value used for SIM, which yielded low noise confocal images; 2) 1.6 microW, which yielded images with much higher noise (S6B Fig, orange line). LSM imaging at 13.5 microW caused substantially faster photobleaching than SIM did, while during LSM imaging at 1.6 microW, photobleaching was slower than in SIM time series.

For nucleoid tracking described below, we acquired 90 s- and 180 s-long SIM time series containing 50 and 100 frames respectively, in SYBR Gold channel only. Immediately before starting the time series, we acquired two-color SIM 3D stacks in SYBG Gold and Mitotracker channels, because our set up did not permit fast enough acquisition of two color channels over time. In the single color time series, we tracked the mitochondrial nucleoids and extracted quantitative parameters of their motions. Most of nucleoids remained apparently at the same positions, showing short-distance random motions apparently confined to mitochondrial network, as one can see from overlay of tracks images on mitochondria images (Fig 9); however, no firm conclusion can be made about the confinement of the tracks within mitochondria, since they were not continuously imaged during tracking time series. Track mean speed was 0.042 μm/s. Only few fast directional displacements per field of view occurred during the observation time window (S1 Video).

Then we aimed to demonstrate that live cell SIM of SYBR Gold-labelled mitochondrial nucleoids can provide information on a biologically relevant effect. Mitochondria are linked to microtubules (e.g. [46]); moreover, it was reported that mitochondrial nucleoids are associated with microtubule network [45]. Thus, here we tested how motions of mitochondrial nucleoids are affected by microtubule depolymerizing drug nocodazole. After nocodazole-induced disassembly of microtubules (confirmed by images of microtubule marker mCherry-MAP4, Fig 10A), we recorded SIM time series of the cells (e.g. S2 Video), detected the tracks of SYBR Gold-stained mitochondrial nucleoids and extracted quantitative track parameters. Results are summarized in Table 2. In the presence of nocodazole, mean track speed distribution and maximal instant track speed distribution shifted towards larger values (Fig 10B–10D); few very long tracks (> 4 μm) appeared (Fig 10C). Interestingly, variation of Instant speed within a track increased from 0.02 to 0.031 upon microtubule depolymerization, showing that motions of nucleoids became more varying (i.e., instant speeds of the nucleoids varied more during

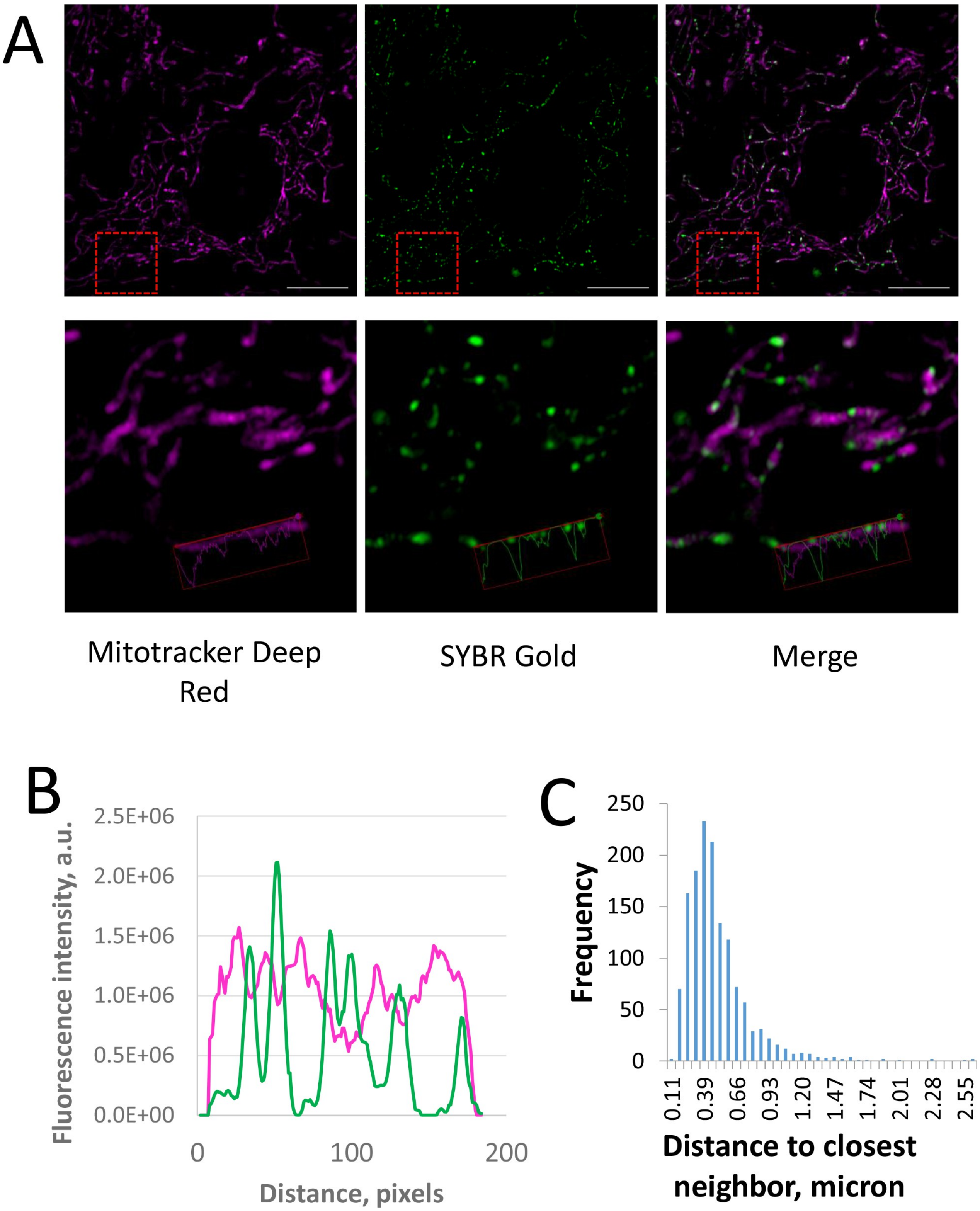

Fig 8. Quantification of mitochondrial nucleoids positions in live HeLa cells on SIM images. A. Representative optical slice of a z-stack of live cells labelled with SYBR Gold (green) and Mitotracker Deep Red (magenta). PS1 Elyra, SIM mode. Red dashed square on the top panels mark a region of interest which is shown on the bottom panels. Scale bar, 10 μm. Intensity profile for a linear ROI is shown on bottom zoomed panels. B. Intensity profile across image of a cell (single optical slice) described above. SYBR Gold signal, green line; Mitotracker Deep Red signal, magenta line. C. Histogram of distribution of distances to the closest neighbors for mitochondrial nucleoids in 3D SIM datasets spanning whole cell thickness.

https://doi.org/10.1371/journal.pone.0203956.g008

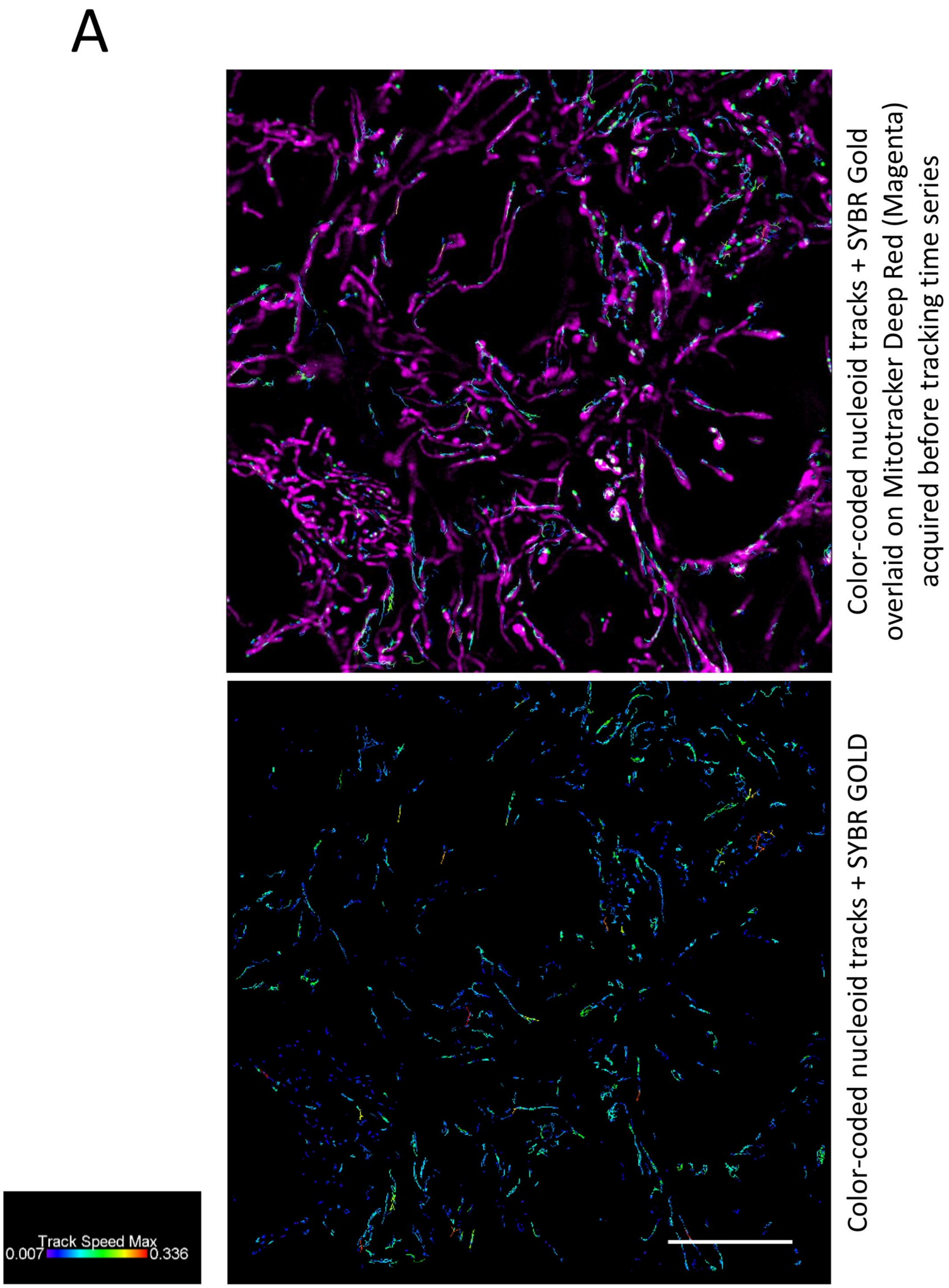

Fig 9. Nucleoid tracking on live SIM images. Representative images of a field of view of DMSO-treated cells. Top: two-color SIM image taken before acquisition of the time lapse series. Green, SYBR Gold channel; magenta, mitotracker channel. Bottom: Nucleoids tracks from 50-frames SIM time series, in SYBR Gold™ channel (S1 and S2 Videos are examples); frame time 1.8 s; tracking by Imaris 8.4.1 software. Tracks are color-coded by maximal track speed (μm/s); PS1 Elyra, SIM mode, 100x/1.46 Oil objective; scale bar 10 μm.

https://doi.org/10.1371/journal.pone.0203956.g009

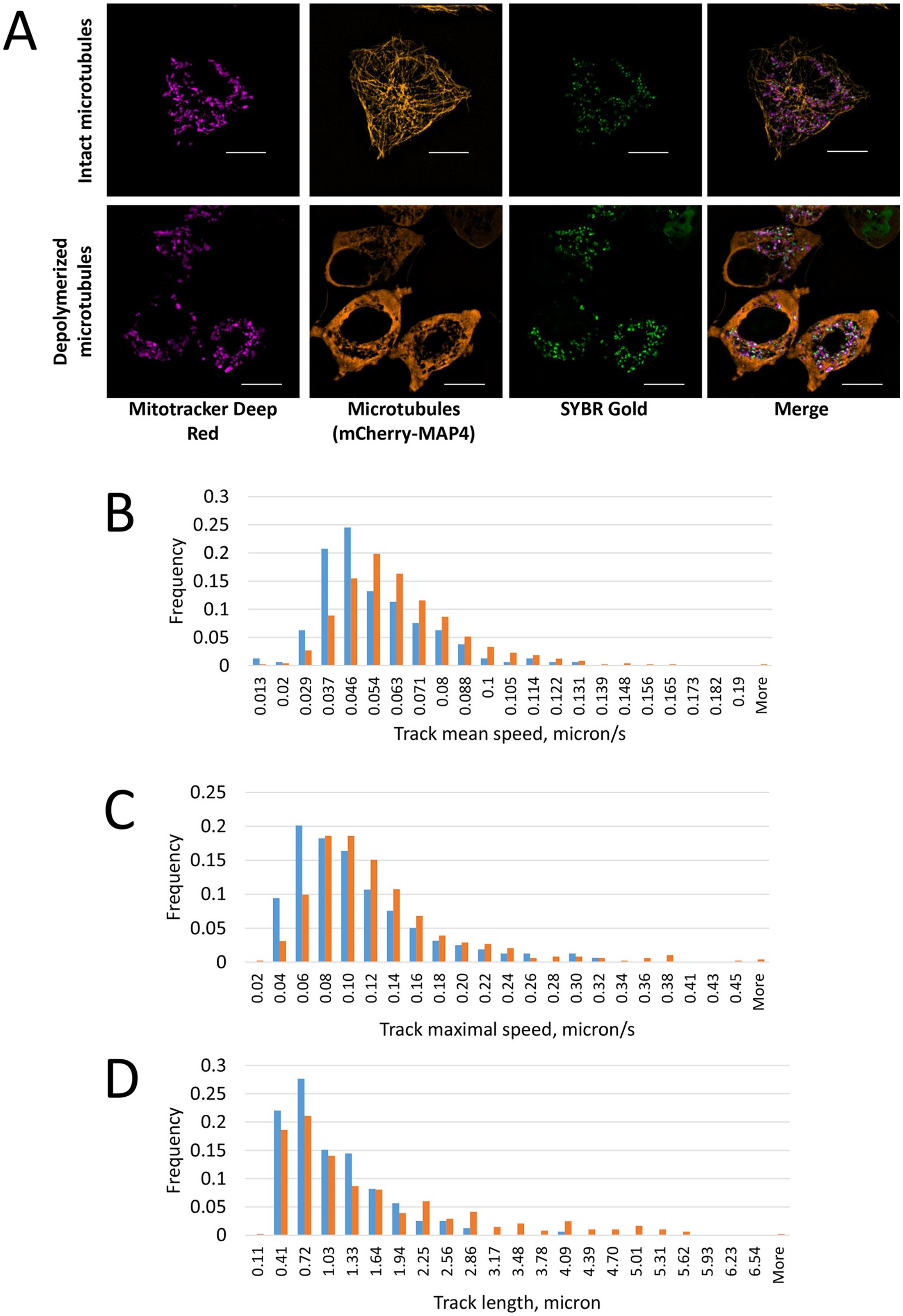
A
Intact microtubules
Depolymerized microtubules
Mitotracker Deep Red
Microtubules (mCherry-MAP4)
SYBR Gold
Merge
B
Frequency
0.3
0.25
0.2
0.15
0.1
0.05
0
0.013 0.02 0.029 0.037 0.046 0.054 0.063 0.071 0.08 0.088 0.1 0.105 0.114 0.122 0.131 0.139 0.148 0.156 0.165 0.173 0.182 0.19 More
Track mean speed, micron/s
C
Frequency
0.25
0.2
0.15
0.1
0.05
0
0.02 0.04 0.06 0.08 0.10 0.12 0.14 0.16 0.18 0.20 0.22 0.24 0.26 0.28 0.30 0.32 0.34 0.36 0.38 0.41 0.43 0.45 More
Track maximal speed, micron/s
D
Frequency
0.3
0.25
0.2
0.15
0.1
0.05
0
0.11 0.41 0.72 1.03 1.33 1.64 1.94 2.25 2.56 2.86 3.17 3.48 3.78 4.09 4.39 4.70 5.01 5.31 5.62 5.93 6.23 6.54 More
Track length, micron

Fig 10. Effect of microtubule depolymerization on the nucleoids motions. A. Control of microtubules depolymerization upon nocodazole treatment. Maximum intensity projections of SIM z-stacks of live HeLa cells stained for mitochondrial DNA (SYBR Gold), mitochondria (Mitotracker Deep Red) and microtubules (transiently expressed MAP4-mCherry construct), without (top) and with (bottom) nocodazole treatment. Scale bar, 10 μm. B-D. Mitochondrial nucleoids tracks parameters in the absence (blue bars) and in the presence (orange bars) of nocodazole. Histograms show distribution of track mean speed (B), track maximal speed (C) and track length (D). Particle tracking is described in Materials and Methods section.

https://doi.org/10.1371/journal.pone.0203956.g010

observation period in the latter case). To explain the observed effects, one has to consider known "anchoring" of mitochondria to microtubule network via mmb1p protein at multiple sites [46] on the one hand, and association of mitochondrial nucleoids with mitochondrial membrane on the other hand [22]. Thus we speculate that nucleoids are indirectly anchored to microtubule network (as it was suggested earlier [45]), and they become more mobile when the network disappears. Obviously, detailed characterization of a putative involvement of microtubules in mitochondrial nucleoids positioning requires further extensive studies, far beyond the scope of the present report.

Discussion and concluding remarks

To get insights into the processes in which mitochondrial nucleoids are involved, one needs a specific, live-cell compatible and convenient labelling combined with a microscopy technique with resolution beyond the diffraction limit ("super-resolution"). Direct labelling of DNA with nucleic acid-binding dyes have several advantages over other labelling strategies. Here, we used this strategy. Based on scientific data, we selected a promising candidate for our application: SYBR Gold has been claimed since long time to be "the most sensitive nucleic acids stain" [42], however, it has never been used for cell labelling, to our best knowledge. We found that SYBR Gold only accumulates in live cells; moreover, this dye efficiently stains nucleoids only in the mitochondria with intact membrane potential; this reduces the risk of subsequent imaging data collection from biologically irrelevant "sick" and dead cells. Tracking of mitochondrial nucleoids at resolution below the diffraction limit and with a modest illumination dose has been achieved for the first time thanks to SYBR Gold staining in combination with SIM, which has been chosen since other major super-resolution techniques, STED and SMLM, require much higher illumination doses, which may cause severe phototoxic effects, especially during time lapse imaging. We observed that at low concentrations SYBR Gold preferentially stained mitochondrial nucleoids, while at high concentrations both chromatin and mtDNA were stained. Interestingly, while this manuscript was in preparation, a similar effect of concentration on subcellular localization has been reported for another positively charged probe

Table 2. Effect of microtubule depolymerization on nucleoids motions parameters.

	Control	Microtubule depolymerization
Track displacement, μm	0.24±0.038	0.27±0.034
Track length, μm	1.06±0.13	1.29±0.11*
Track maximal instant speed, μm/s	0.078±0.012	0.123±0.008*
Track mean speed, μm/s	0.042±0.005	0.064±0.004*
Variation of Instant Speed within a Track	0.020±0.003	0.031±0.001*
Track straightness	0.33±0.044	0.28±0.017

Quantitative parameters of the tracks were obtained by Imaris 8.4.1 software, as described in Materials and Methods. Student t-test was applied to average values calculated for each field of view (n = 4. . .6).

*Significant difference between the control and nocodazole treatment, $P<0.05$.

https://doi.org/10.1371/journal.pone.0203956.t002

I^{4+}, which has no structural similarity to SYBR Gold—[47]: the probe accumulated in the live nuclei at a high concentration, while it localized in the mitochondria at a low concentration; however, no targeting to nucleoids appeared, in contrast our observations on SYBR Gold. Unfortunately, mitochondrial nucleoids staining with SYBR Gold is not compatible with conventional IF protocols because it does not resist permeabilization step. However, to label nucleoids in fixed cells, other good tools exist, such as antibodies against DNA and TFAM.

We expect that SYBR Gold will be widely used of for live imaging of mitochondrial nucleoids. Our study of SYBR Gold emphasizes that other organic dyes widely used *in vitro* may be useful for fluorescence microscopy or other cell-based technique like flow cytometry. In general, present study illustrates usefulness of "re-targeting" of known molecules, a strategy which is easier and faster than rational design of novel labels from scratch.

Supporting information

S1 Video. Effect of microtubules depolymerization on mitochondrial nucleoids motions. SIM time lapse series showing representative SYBR Gold-stained cells without nocodazole treatment. Scale bar, 5 μm. Detected mitochondrial nucleoids are marked as white balls. Tracks are visualized as "Dragon tails" (8 frames length) and color-coded according to their maximal instant speeds (color bar in the bottom right shows speeds in μm/s). Mitochondrial nucleoids tracking and visualization by Imaris 8.4.1 software.
(AVI)

S2 Video. Effect of microtubules depolymerization on mitochondrial nucleoids motions. SIM time lapse series showing representative SYBR Gold-stained cells with nocodazole treatment. Scale bar, 5 μm. Detected mitochondrial nucleoids are marked as white balls. Tracks are visualized as "Dragon tails" (8 frames length) and color-coded according to their maximal instant speeds (color bar in the bottom right shows speeds in μm/s). Mitochondrial nucleoids tracking and visualization by Imaris 8.4.1 software.
(AVI)

S1 Fig. SYBR Gold localization in live cells upon labelling at different concentrations. HeLa cells were incubated for 30 min with mixture of 0.25 μM Mitotracker CMXRos Red and indicated SYBR Gold dilution; the solution was replaced with DMEM and images were acquired on LSM880 Airyscan microscope, 63x 1.4 oil objective, sequential acquisition of color channels; Single optical slices are shown; scale bar 10 μm.
(TIF)

S2 Fig. HeLa cells during labelling with SYBR Gold. First, live HeLa cells were labelled with Mitotracker CMXRos Red and washed; then SYBR Gold (final dilution 1:10000 in DMEM) was added to the cells and time lapse acquisition has been started. LSM880 microscope, 63x 1.4 Oil objective, sequential acquisition. Z-stacks were acquired at each time point; maximum intensity projections are shown. A. Representative fields of view showing the regions of interest where SYBR Gold fluorescence was measured (colored rectangles). B. Mean intensities of SYBR Gold fluorescence over time in the regions of interest shown on S2A Fig; curve colors correspond to the rectangles on S2A Fig. C. A field of view at several time points during incubation with SYBR Gold. A square region is shown (marked with white line) with higher magnification in the right column.
(TIF)

S3 Fig. Co-localisation of TFAM and SYBR Gold staining in HEK-T cells. Live HEK-T cells; LSM780 confocal microscope, 63x/1.4 Oil objective; 3D image stacks were acquired and used

for co-localization analysis. Single optical slice is shown; scale bar 10 μm. Signals in Mitotracker Deep Red (white), TFAM-mEos2 (red) and SYBR Gold (green) channels were acquired sequentially, with switching channels every scanned line. 18 fields of view from two independent transfection experiments were acquired. A representative field of view is shown. Red dashed squares on the left panels mark the region of interest which is shown at higher magnification in the right panels.
(PDF)

S4 Fig. Localization of anti-TFAM antibody in live cells stained with SYBG Gold. Live HeLa cells were transfected with anti-TFAM antibody conjugated to PF555 dye and then stained for 30 min. with SYBR Gold (final dilution 1:10000) and Mitotracker Deep Red™ (final concentration 250 nM). Images (z-stacks) of live cells were acquired on Zeiss LSM780 microscope with 63x/1.4 Oil objective; channels were acquired sequentially; detection ranges were adjusted to minimize spectral bleed-through: (500–550 nm for SYBR Gold, 565–598 nm for PF555 and 645–700 nm for Mitotracker Deep Red. Deconvolution of datasets was performed. Single optical slice from a representative field of view is shown; scale bar 10 μm. Cyan squares on the left panels mark the region of interest which is shown at a higher magnification in the right panels.
(PDF)

S5 Fig. Live cells stained with Hoechst 33342 and propidium iodide, 2 days after incubation with SYBR Gold. A representative 1.19×1.19 mm field of view used for calculation of the portion of dead cells (Table 1). Maximum intensity projection of a z-stack is shown. Scale bar 100 μm.
(PDF)

S6 Fig. Comparison of acquisition photobleaching in confocal and SIM modes. A. Time lapse series of live HeLa cells labelled with SYBR Gold. SIM settings are described in Materials and Methods section. Briefly, frame time 1.8 s, 488 nm laser, 1% AOTF (corresponding to 13.5 microW, 0.54 mW/mm^2). Confocal time series were acquired for the same field of view (50 by 50 μm), the same pixel size (50 nm) and same frame time (1.8 s) as for SIM images. Confocal imaging was performed under two settings: 1) blue, the same illumination power as for SIM (13.5 microW); 2) orange, illumination power reduced to 1.6 microW. B. Intensity profiles across nucleoids on confocal images acquired with 13.5 microW (blue) and 1.6 microW (orange).
(PDF)

Acknowledgments

The authors thank Nathalie Daigle and Jan Ellenberg (EMBL Heidelberg) for mCherry-MAP4 plasmid, and Tim Brown (Howard Hughes Medical Institute, Ashburn, VA) for TFAM-mEos2 plasmid. Authors acknowledge Asifa Akhtar and Angelika Rambold (both Max Planck Institute for Immunobiology and Epigenetics) for providing HeLa cells.

Author Contributions

Conceptualization: Sergiy V. Avilov.

Data curation: Visnja Jevtic.

Formal analysis: Sergiy V. Avilov.

Funding acquisition: Sergiy V. Avilov.

Investigation: Visnja Jevtic, Petra Kindle, Sergiy V. Avilov.

Methodology: Sergiy V. Avilov.

Project administration: Sergiy V. Avilov.

Supervision: Sergiy V. Avilov.

Visualization: Sergiy V. Avilov.

Writing – original draft: Sergiy V. Avilov.

Writing – review & editing: Visnja Jevtic, Sergiy V. Avilov.

References

1. Gilkerson RW, De Vries RL, Lebot P, Wikstrom JD, Torgyekes E, Shirihai OS, et al. Mitochondrial autophagy in cells with mtDNA mutations results from synergistic loss of transmembrane potential and mTORC1 inhibition. Hum Mol Genet. 2012; 21(5):978–90. https://doi.org/10.1093/hmg/ddr529 PMID: 22080835; PubMed Central PMCID: PMCPMC3277306.
2. Wu Z, Puigserver P, Andersson U, Zhang C, Adelmant G, Mootha V, et al. Mechanisms controlling mitochondrial biogenesis and respiration through the thermogenic coactivator PGC-1. Cell. 1999; 98 (1):115–24. https://doi.org/10.1016/S0092-8674(00)80611-X PMID: 10412986.
3. Chatterjee A, Seyfferth J, Lucci J, Gilsbach R, Preissl S, Bottinger L, et al. MOF Acetyl Transferase Regulates Transcription and Respiration in Mitochondria. Cell. 2016; 167(3):722–38 e23. https://doi.org/10.1016/j.cell.2016.09.052 PMID: 27768893.
4. Kang D, Kim SH, Hamasaki N. Mitochondrial transcription factor A (TFAM): roles in maintenance of mtDNA and cellular functions. Mitochondrion. 2007; 7(1–2):39–44. https://doi.org/10.1016/j.mito.2006.11.017 PMID: 17280879.
5. Bogenhagen DF. Mitochondrial DNA nucleoid structure. Biochim Biophys Acta. 2012; 1819(9–10):914–20. https://doi.org/10.1016/j.bbagrm.2011.11.005 PMID: 22142616.
6. Kaufman BA, Durisic N, Mativetsky JM, Costantino S, Hancock MA, Grutter P, et al. The mitochondrial transcription factor TFAM coordinates the assembly of multiple DNA molecules into nucleoid-like structures. Mol Biol Cell. 2007; 18(9):3225–36. https://doi.org/10.1091/mbc.E07-05-0404 PMID: 17581862; PubMed Central PMCID: PMCPMC1951767.
7. Farge G, Laurens N, Broekmans OD, van den Wildenberg SM, Dekker LC, Gaspari M, et al. Protein sliding and DNA denaturation are essential for DNA organization by human mitochondrial transcription factor A. Nature communications. 2012; 3:1013. https://doi.org/10.1038/ncomms2001 PMID: 22910359.
8. Alam TI, Kanki T, Muta T, Ukaji K, Abe Y, Nakayama H, et al. Human mitochondrial DNA is packaged with TFAM. Nucleic Acids Res. 2003; 31(6):1640–5. PMID: 12626705; PubMed Central PMCID: PMCPMC152855.
9. Pernas L, Scorrano L. Mito-Morphosis: Mitochondrial Fusion, Fission, and Cristae Remodeling as Key Mediators of Cellular Function. Annu Rev Physiol. 2016; 78:505–31. https://doi.org/10.1146/annurev-physiol-021115-105011 PMID: 26667075.
10. Tauber J, Dlaskova A, Santorova J, Smolkova K, Alan L, Spacek T, et al. Distribution of mitochondrial nucleoids upon mitochondrial network fragmentation and network reintegration in HEPG2 cells. Int J Biochem Cell Biol. 2013; 45(3):593–603. https://doi.org/10.1016/j.biocel.2012.11.019 PMID: 23220174.
11. Dlaskova A, Engstova H, Plecita-Hlavata L, Lessard M, Alan L, Reguera DP, et al. Distribution of mitochondrial DNA nucleoids inside the linear tubules vs. bulk parts of mitochondrial network as visualized by 4Pi microscopy. J Bioenerg Biomembr. 2015; 47(3):255–63. https://doi.org/10.1007/s10863-015-9610-3 PMID: 25833036.
12. Garrido N, Griparic L, Jokitalo E, Wartiovaara J, van der Bliek AM, Spelbrink JN. Composition and dynamics of human mitochondrial nucleoids. Mol Biol Cell. 2003; 14(4):1583–96. https://doi.org/10.1091/mbc.E02-07-0399 PMID: 12686611; PubMed Central PMCID: PMCPMC153124.
13. Legros F, Malka F, Frachon P, Lombes A, Rojo M. Organization and dynamics of human mitochondrial DNA. J Cell Sci. 2004; 117(Pt 13):2653–62. https://doi.org/10.1242/jcs.01134 PMID: 15138283.
14. Caielli S, Athale S, Domic B, Murat E, Chandra M, Banchereau R, et al. Oxidized mitochondrial nucleoids released by neutrophils drive type I interferon production in human lupus. J Exp Med. 2016; 213 (5):697–713. https://doi.org/10.1084/jem.20151876 PMID: 27091841; PubMed Central PMCID: PMCPMC4854735.

15. Okayama S, Ohta K, Higashi R, Nakamura K. Correlative light and electron microscopic observation of mitochondrial DNA in mammalian cells by using focused-ion beam scanning electron microscopy. Microscopy (Oxf). 2014; 63 Suppl 1:i35. https://doi.org/10.1093/jmicro/dfu079 PMID: 25359841.

16. Alan L, Spacek T, Jezek P. Delaunay algorithm and principal component analysis for 3D visualization of mitochondrial DNA nucleoids by Biplane FPALM/dSTORM. Eur Biophys J. 2016; 45(5):443–61. https://doi.org/10.1007/s00249-016-1114-5 PMID: 26846371.

17. Kukat C, Wurm CA, Spahr H, Falkenberg M, Larsson NG, Jakobs S. Super-resolution microscopy reveals that mammalian mitochondrial nucleoids have a uniform size and frequently contain a single copy of mtDNA. Proc Natl Acad Sci U S A. 2011; 108(33):13534–9. https://doi.org/10.1073/pnas.1109263108 PMID: 21808029; PubMed Central PMCID: PMCPMC3158146.

18. Kukat C, Larsson NG. mtDNA makes a U-turn for the mitochondrial nucleoid. Trends Cell Biol. 2013; 23 (9):457–63. https://doi.org/10.1016/j.tcb.2013.04.009 PMID: 23721879.

19. Hell SW, Wichmann J. Breaking the diffraction resolution limit by stimulated emission: stimulated-emission-depletion fluorescence microscopy. Opt Lett. 1994; 19(11):780–2. PMID: 19844443.

20. Betzig E, Patterson GH, Sougrat R, Lindwasser OW, Olenych S, Bonifacino JS, et al. Imaging intracellular fluorescent proteins at nanometer resolution. Science. 2006; 313(5793):1642–5. https://doi.org/10.1126/science.1127344 PMID: 16902090.

21. Rust MJ, Bates M, Zhuang X. Sub-diffraction-limit imaging by stochastic optical reconstruction microscopy (STORM). Nat Methods. 2006; 3(10):793–5. https://doi.org/10.1038/nmeth929 PMID: 16896339; PubMed Central PMCID: PMCPMC2700296.

22. Brown TA, Tkachuk AN, Shtengel G, Kopek BG, Bogenhagen DF, Hess HF, et al. Superresolution fluorescence imaging of mitochondrial nucleoids reveals their spatial range, limits, and membrane interaction. Mol Cell Biol. 2011; 31(24):4994–5010. https://doi.org/10.1128/MCB.05694-11 PMID: 22006021; PubMed Central PMCID: PMCPMC3233019.

23. Kukat C, Davies KM, Wurm CA, Spahr H, Bonekamp NA, Kuhl I, et al. Cross-strand binding of TFAM to a single mtDNA molecule forms the mitochondrial nucleoid. Proc Natl Acad Sci U S A. 2015; 112 (36):11288–93. https://doi.org/10.1073/pnas.1512131112 PMID: 26305956; PubMed Central PMCID: PMCPMC4568684.

24. Benke A, Manley S. Live-cell dSTORM of cellular DNA based on direct DNA labeling. Chembiochem. 2012; 13(2):298–301. https://doi.org/10.1002/cbic.201100679 PMID: 22213360.

25. Ishigaki M, Iketani M, Sugaya M, Takahashi M, Tanaka M, Hattori S, et al. STED super-resolution imaging of mitochondria labeled with TMRM in living cells. Mitochondrion. 2016; 28:79–87. https://doi.org/10.1016/j.mito.2016.03.009 PMID: 27090168.

26. Waldchen S, Lehmann J, Klein T, van de Linde S, Sauer M. Light-induced cell damage in live-cell super-resolution microscopy. Sci Rep. 2015; 5:15348. https://doi.org/10.1038/srep15348 PMID: 26481189; PubMed Central PMCID: PMCPMC4611486.

27. Gustafsson MG, Shao L, Carlton PM, Wang CJ, Golubovskaya IN, Cande WZ, et al. Three-dimensional resolution doubling in wide-field fluorescence microscopy by structured illumination. Biophys J. 2008; 94(12):4957–70. https://doi.org/10.1529/biophysj.107.120345 PMID: 18326650; PubMed Central PMCID: PMCPMC2397368.

28. Hirano Y, Matsuda A, Hiraoka Y. Recent advancements in structured-illumination microscopy toward live-cell imaging. Microscopy (Oxf). 2015; 64(4):237–49. https://doi.org/10.1093/jmicro/dfv034 PMID: 26133185.

29. Langhorst MF, Schaffer J, Goetze B. Structure brings clarity: structured illumination microscopy in cell biology. Biotechnol J. 2009; 4(6):858–65. https://doi.org/10.1002/biot.200900025 PMID: 19492328.

30. Lewis SC, Uchiyama LF, Nunnari J. ER-mitochondria contacts couple mtDNA synthesis with mitochondrial division in human cells. Science. 2016; 353(6296):aaf5549. https://doi.org/10.1126/science.aaf5549 PMID: 27418514.

31. Cranfill PJ, Sell BR, Baird MA, Allen JR, Lavagnino Z, de Gruiter HM, et al. Quantitative assessment of fluorescent proteins. Nat Methods. 2016; 13(7):557–62. https://doi.org/10.1038/nmeth.3891 PMID: 27240257; PubMed Central PMCID: PMCPMC4927352.

32. Marx V. Probes: paths to photostability. Nat Methods. 2015; 12(3):187–90. https://doi.org/10.1038/nmeth.3295 PMID: 25719826.

33. Dellinger M, Geze M. Detection of mitochondrial DNA in living animal cells with fluorescence microscopy. J Microsc. 2001; 204(Pt 3):196–202. PMID: 11903796.

34. Ban-Ishihara R, Ishihara T, Sasaki N, Mihara K, Ishihara N. Dynamics of nucleoid structure regulated by mitochondrial fission contributes to cristae reformation and release of cytochrome c. Proc Natl Acad Sci U S A. 2013; 110(29):11863–8. https://doi.org/10.1073/pnas.1301951110 PMID: 23821750; PubMed Central PMCID: PMCPMC3718159.

35. Zurek-Biesiada D, Szczurek AT, Prakash K, Mohana GK, Lee HK, Roignant JY, et al. Localization microscopy of DNA in situ using Vybrant((R)) DyeCycle Violet fluorescent probe: A new approach to study nuclear nanostructure at single molecule resolution. Exp Cell Res. 2016; 343(2):97–106. https://doi.org/10.1016/j.yexcr.2015.08.020 PMID: 26341267.

36. Zurek-Biesiada D, Szczurek AT, Prakash K, Best G, Mohana GK, Lee HK, et al. Quantitative super-resolution localization microscopy of DNA in situ using Vybrant(R) DyeCycle Violet fluorescent probe. Data Brief. 2016; 7:157–71. https://doi.org/10.1016/j.dib.2016.01.041 PMID: 27054149; PubMed Central PMCID: PMCPMC4802433.

37. Bereiter-Hahn J, Vöth M. Distribution and dynamics of mitochondrial nucleoids in animal cells in culture. EBO—Experimental Biology Online Annual 1996/97. Berlin, Heidelberg: Springer Berlin Heidelberg; 1998. p. 58–77.

38. He J, Mao CC, Reyes A, Sembongi H, Di Re M, Granycome C, et al. The AAA+ protein ATAD3 has displacement loop binding properties and is involved in mitochondrial nucleoid organization. J Cell Biol. 2007; 176(2):141–6. https://doi.org/10.1083/jcb.200609158 PMID: 17210950; PubMed Central PMCID: PMCPMC2063933.

39. Ashley N, Harris D, Poulton J. Detection of mitochondrial DNA depletion in living human cells using PicoGreen staining. Exp Cell Res. 2005; 303(2):432–46. https://doi.org/10.1016/j.yexcr.2004.10.013 PMID: 15652355.

40. Chen LB. Mitochondrial membrane potential in living cells. Annu Rev Cell Biol. 1988; 4:155–81. https://doi.org/10.1146/annurev.cb.04.110188.001103 PMID: 3058159.

41. Negwer I, Hirsch M, Kaloyanova S, Brown T, Peneva K, Butt HJ, et al. Modulation of Mitochondriotropic Properties of Cyanine Dyes by in Organello Copper-Free Click Reaction. Chembiochem. 2017. https://doi.org/10.1002/cbic.201700286 PMID: 28704573.

42. Tuma RS, Beaudet MP, Jin X, Jones LJ, Cheung CY, Yue S, et al. Characterization of SYBR Gold nucleic acid gel stain: a dye optimized for use with 300-nm ultraviolet transilluminators. Anal Biochem. 1999; 268(2):278–88. https://doi.org/10.1006/abio.1998.3067 PMID: 10075818.

43. Keppler A, Ellenberg J. Chromophore-assisted laser inactivation of alpha- and gamma-tubulin SNAP-tag fusion proteins inside living cells. ACS Chem Biol. 2009; 4(2):127–38. https://doi.org/10.1021/cb800298u PMID: 19191588.

44. Carpenter AE, Jones TR, Lamprecht MR, Clarke C, Kang IH, Friman O, et al. CellProfiler: image analysis software for identifying and quantifying cell phenotypes. Genome Biol. 2006; 7(10):R100. https://doi.org/10.1186/gb-2006-7-10-r100 PMID: 17076895; PubMed Central PMCID: PMCPMC1794559.

45. Iborra FJ, Kimura H, Cook PR. The functional organization of mitochondrial genomes in human cells. BMC Biol. 2004; 2:9. https://doi.org/10.1186/1741-7007-2-9 PMID: 15157274; PubMed Central PMCID: PMCPMC425603.

46. Fu C, Jain D, Costa J, Velve-Casquillas G, Tran PT. mmb1p binds mitochondria to dynamic microtubules. Curr Biol. 2011; 21(17):1431–9. https://doi.org/10.1016/j.cub.2011.07.013 PMID: 21856157; PubMed Central PMCID: PMCPMC4720976.

47. Sreedharan S, Gill MR, Garcia E, Saeed HK, Robinson D, Byrne A, et al. Multimodal Super-resolution Optical Microscopy Using a Transition-Metal-Based Probe Provides Unprecedented Capabilities for Imaging Both Nuclear Chromatin and Mitochondria. J Am Chem Soc. 2017; 139(44):15907–13. https://doi.org/10.1021/jacs.7b08772 PMID: 28976195.
